# DNA methylation-driven EMT is a common mechanism of resistance to various therapeutic agents in cancer

**DOI:** 10.1186/s13148-020-0821-z

**Published:** 2020-02-14

**Authors:** Eva Galle, Bernard Thienpont, Sarah Cappuyns, Tom Venken, Pieter Busschaert, Matthias Van Haele, Eric Van Cutsem, Tania Roskams, Jos van Pelt, Chris Verslype, Jeroen Dekervel, Diether Lambrechts

**Affiliations:** 1 Centre for Cancer Biology, VIB, 3000 Leuven, Belgium; 2 Laboratory of Translational Genetics, Department of Human Genetics, KU Leuven, 3000 Leuven, Belgium; 3 Laboratory for Functional Epigenetics, Department of Human Genetics, KU Leuven, 3000 Leuven, Belgium; 4 Clinical Digestive Oncology, Department of Oncology, KU Leuven and University Hospitals Leuven, 3000 Leuven, Belgium; 5 Department of Imaging and Pathology, Translational Cell and Tissue Research, KU Leuven and University Hospitals Leuven, 3000 Leuven, Belgium; 6 Laboratory of Clinical Digestive Oncology, Department of Oncology, KU Leuven and Leuven Cancer Institute (LKI), 3000 Leuven, Belgium

**Keywords:** Cancer, DNA methylation, Epithelial-to-mesenchymal transition, Liquid biopsies, Therapy resistance

## Abstract

**Background:**

Overcoming therapeutic resistance is one of the major hurdles in cancer care. One mechanism contributing to therapeutic resistance is a process in which epithelial cells switch to a mesenchymal state (epithelial-to-mesenchymal transition or EMT). The precise mechanisms driving EMT-mediated therapeutic resistance have, however, not been elucidated.

**Results:**

Here, we study ten cell line pairs, for which parental cell lines were made resistant to either a targeted or chemotherapy-based treatment. First, we show by miRNA-200 overexpression that treatment resistance is driven by EMT. Next, we demonstrate that DNA methylation changes occur within each cell line pair and show that exposure to 5-azacytidine or knock down of DNA methyltransferases (DNMTs), both of which globally demethylate cells, result in EMT reversal and increased therapeutic sensitivity. This suggests DNA methylation to causally underlie EMT and treatment resistance. We also observe significant overlap in methylation profiles between resistant lines, suggesting a common epigenetic mechanism to cause resistance to therapy. In line with this hypothesis, cross-resistance to other targeted and chemotherapies is observed, while importantly, this is lost upon demethylation of the cells. Finally, we clinically validate that DNA methylation changes drive EMT-mediated resistance to sorafenib in patients with advanced hepatocellular carcinoma (HCC). Specifically, we develop a capture-based protocol to interrogate DNA methylation in low amounts of circulating tumor DNA (ctDNA). By interrogating the methylation status in liquid biopsies, longitudinally collected during sorafenib treatment, we assess whether DNA methylation changes also drive EMT and therapy resistance in a clinical setting. Particularly, by monitoring methylation changes in EMT genes, we are able to predict tumor response and acquired resistance to sorafenib.

**Conclusions:**

We propose methylation changes underlying EMT to constitute a common resistance mechanism to cancer therapies. This process can be reversed pharmacologically and monitored non-invasively in ctDNA to predict resistance to treatment.

## Background

The epithelial-to-mesenchymal transition (EMT) is a highly dynamic process, by which epithelial cells convert into a mesenchymal phenotype [1]. In tumors, EMT is implicated in enhancing the cellular motility and metastatic propensity of the cancer cells [2]. Additionally, cancer cells undergoing EMT tend to lose sensitivity to a variety of anti-cancer treatments [3, 4]. Despite its manifested clinical importance, the mechanisms underlying EMT-mediated resistance to cancer therapies have not been systematically assessed [5].

Indeed, although some studies already reported that the phenotypic alterations underlying EMT are accompanied by epigenetic changes [1, 6, 7], these studies often focus on the epigenetic state of only a single EMT gene, while many different epithelial and mesenchymal genes are actually known to act in concert during the process. For instance, gene promoter DNA methylation of the main EMT-inducing transcription factors, *TWIST1*/*2*, *ZEB2*, and *SNAI1*/*2* [8–10], but also the promoter of the gene encoding E-cadherin, a prototypical epithelial marker, have each been reported to be differentially methylated following EMT [8, 11–14]. Another shortcoming is that most of these studies use transformed cell lines, in which EMT is artificially induced via forced overexpression of genes involved in EMT, such as transforming growth factor-beta (TGF-B)*.* Such artificial systems, however, do not faithfully reflect the regulation of EMT genes under pathophysiological conditions. Finally, for most studies, it is unclear whether the observed epigenetic changes are causally underlying the observed EMT phenotype, or whether they are merely a consequence of it.

DNA methylation represents one of the best characterized mechanisms of epigenetic gene regulation and might indeed be involved in regulating EMT. We have previously shown that tumor hypoxia, a known inducer of EMT, can directly impair the activity of ten-eleven translocation (TET) DNA demethylases by reducing the availability of oxygen, an essential cofactor of TET enzymes [15]. The resultant decrease in DNA demethylation activity led to an accumulation of DNA methylation which, while mostly occurring near tumor suppressor genes, also affected genes encoding cell adhesion functions that are involved in the EMT process [15]. We therefore investigated here whether DNA methylation changes contribute to EMT-mediated resistance to various therapies, in cell lines originating from hepatocellular and pancreatic carcinoma, as well as from lung and ovarian cancer, and used pharmacological and siRNA-mediated DNA demethylation to establish its causality in maintaining a mesenchymal phenotype. We hypothesize that altered TET activity during exposure to chemo- or targeted therapies causes DNA methylation changes that dampen the epithelial gene expression program, while increasing expression of mesenchymal genes, thereby contributing to EMT and therapy resistance.

Most importantly, it has never been explored to what extent DNA methylation and EMT also mediate therapy resistance in cancer patients, thus raising questions about the clinical and diagnostic relevance [1, 5]. In an effort to better understand this, we developed an innovative cell-free DNA-based assay capable of interrogating EMT-associated circulating tumor DNA (ctDNA) methylation changes in serially collected liquid biopsies of advanced hepatocellular cancer (HCC) patients receiving sorafenib as first-line therapy. For the first time, this allowed us to monitor to what extent DNA methylation-driven EMT also occurs in cancer patients developing acquired resistance to their cancer treatment.

## Results

### Treatment-naïve and treatment-resistant paired cell lines

To explore whether epigenetic changes underlie resistance to cancer therapies, we from literature identified treatment-naïve epithelial cancer cell lines (hereafter referred to as parental cell lines) that were rendered resistant to a cancer treatment (hereafter referred to as treatment-resistant lines) by exposing the cells to gradually increasing treatment doses over the course of several months. We specifically looked for reports in which no established resistance mechanism was described and/or detected (such as mutations or amplifications in drug target or multidrug resistance genes), but in which changes reminiscent of EMT were associated with the underlying drug resistance phenotype. Overall, we retrieved five cell line pairs of lung and pancreatic adenocarcinoma and five cell line pairs of hepatocellular and ovarian carcinoma, either exposed to targeted cancer therapies (sorafenib, gefitinib, erlotinib, and olaparib) or chemotherapy (5-fluorouracil, cisplatin; Table 1).

**Table 1:** Resistant cell lines

Parental cell line	RRID	Origin	Therapy drug	Parental IC_50_	Therapy target	Exposure period	Resistant cell line	Resistant IC_50_	EMT	Ref
HepG2 (*n* = 2)	CVCL_0027	Liver	Sorafenib	0.28 μM	VEGFR, PDGFR, Raf kinases	6–12 months	HepG2S1	0.44 μM	Full	[16]
							HepG2S3	0.69 μM	Full	
HCC4006P1	CVCL_1269	Lung	Erlotinib	0.03 μM	EGFR	3–6 months	HCC4006ER	0.97 μM	Full	[17]
Panc 03.27 (‘PancVB’) (*n* = 2)	CVCL_1635	Pancreas	5-Fluorouracil	14.63 μM	Thymidylate synthase	6 months	PancB1Q	55.72 μM	Full	[18]
							PancB1V	30.75 μM	Full	
HCC4006P2	CVCL_1269	Lung	Gefitinib	0.10 μM	EGFR	3 months	HCC4006GR	0.59 μM	Full	[19]
HCC827P	CVCL_2063	Lung	Gefitinib	0.027 μM	EGFR	3 months	HCC827GR	0.046 μM	Full	
UWB 1.289 (*n* = 2)	CVCL_B079	Ovary	Olaparib	58.02 μM	PARP	5 months	U10	512.33 μM	Partial	[20]
							U100	699.41 μM	Partial	
IGROV-1	CVCL_1304	Ovary	Cisplatin	64.04 μM	DNA replication	9 months	IGROV-1/CDDP	388.02 μM	Partial	[21]

The columns indicate which established cell line was used as the parental cell line, their Research Resource Identifiers (RRID), their tissue of origin, the drug they were made resistant to, the concentration of the drugs at which their growth was inhibited by 50% (IC_50_), the target of the drug, the period over which they were rendered resistant, the name of their resistant counterparts, the concentration of drugs at which the growth of the resistant cells was inhibited by 50% (IC_50_), their EMT status and the reference of the paper describing their retrieval

**Fig. 1:**
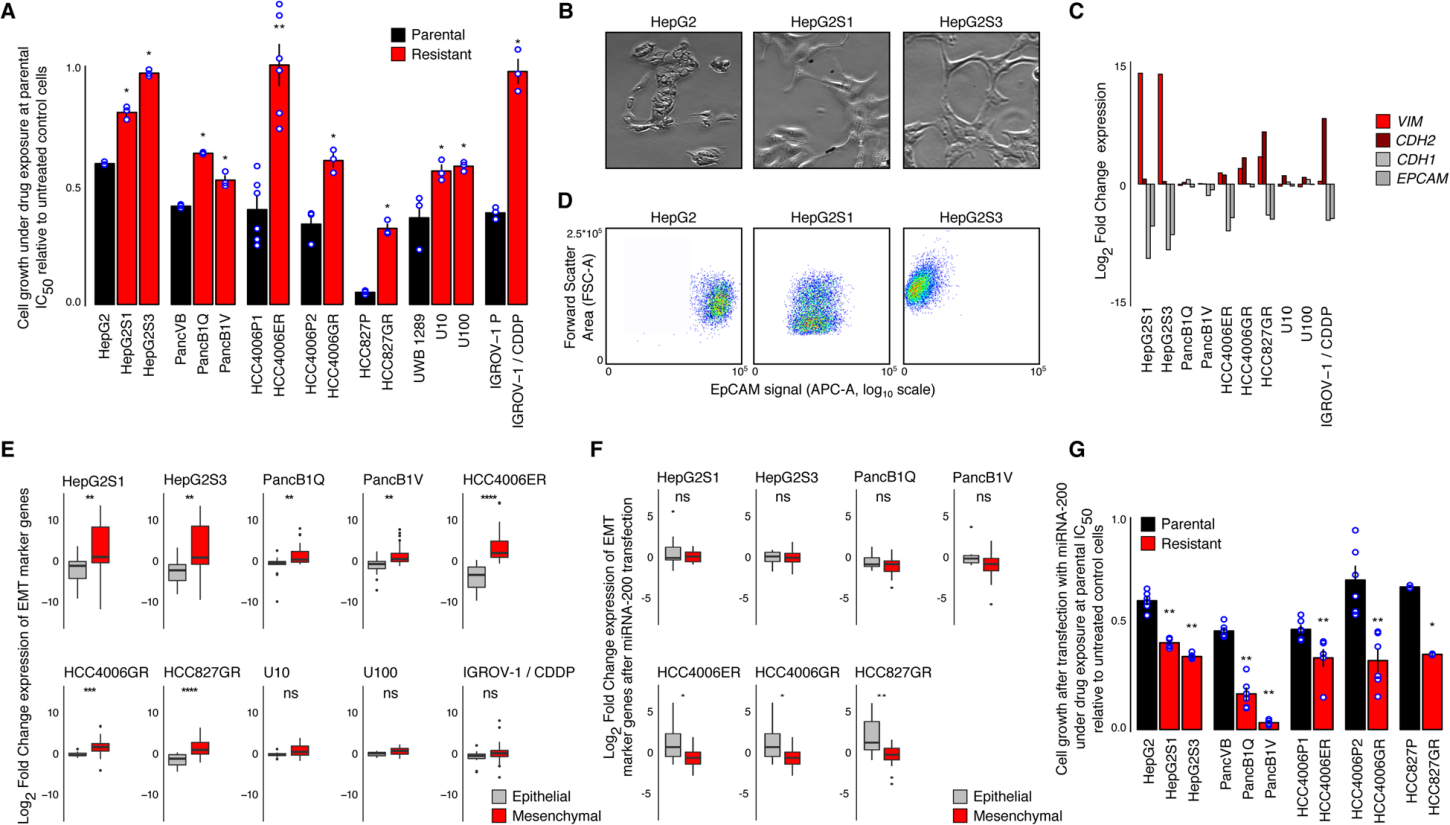
Cell lines serving as models for EMT-mediated resistance. **a** Growth of parental (black) and resistant (red) cells upon exposure to the drugs they were rendered resistant to, at concentrations estimated to inhibit the growth of parental cells by 50% (IC_50_, Table 1). Growth is calculated relative to cells unexposed to the drugs. Shown are individual data points and means of experiments performed in triplicate (except for the pair HCC4006P1 and HCC4006ER, *n* = 6), each replication consisting of nine technical replicates. Significance of the difference between parental and resistant cell growth is according to a one-sided Mann-Whitney *U* test. **b** Example of morphological changes associated with EMT, showing a parental epithelial cell line (HepG2) and two derived resistant mesenchymal cell lines (HepG2S1 and HepG2S3). **c** Expression differences (log_2_ fold-change) of common EMT markers, vimentin (*VIM*), N-cadherin (*CDH2*), E-cadherin (*CDH1*), and epithelial cell adhesion molecule (*EPCAM*), between resistant and parental cells. Names of resistant lines indicate cell line pairs. **d** Cell surface expression of EPCAM protein in a parental (HepG2) and two resistant cell lines (HepG2S1 and HepG2S3), measured using flow cytometry. Shown are signal intensities of APC-conjugated anti-EPCAM antibody (*x*-axis, log_10_ scale) and forward scatter area (FSC-A; *y*-axis, linear scale). **e** Expression changes in EMT marker genes following resistance acquisition. Boxplots illustrate expression differences (log_2_ fold-change) between resistant and parental cells of 16 epithelial (gray) and 34 mesenchymal (red) marker genes (Additional file 1: Table S1), determined using RNA sequencing. Significance of differences between epithelial and mesenchymal marker gene expression changes was calculated using a two-sided Mann-Whitney *U* test. **f** As in panel (E) for cells transfected with miRNA-200, determined using RT-qPCR. **g** As in panel (**a**) for cells transfected with miRNA-200, relative to miRNA-200 transfected cells unexposed to the drugs. For all cell line pairs: *n* = 6, except for HCC827P and HCC827GR: *n* = 3. *ns* non-significant, *p value < 0.05, **p value < 0.01, ***p value < 0.001, ****p value < 0.0001

First, we confirmed that parental cell lines were more sensitive to their respective treatment than treatment-resistant cell lines. For this, we calculated the drug concentration that inhibits cell growth by 50% (i.e., IC_50_) using the Sulforhodamine B (SRB) colorimetric assay [22] both for the parental and treatment-resistant cell lines. This analysis confirmed that treatment-resistant cells were characterized by higher inhibitory concentration 50 (IC_50_) values than their parental counterparts (Additional file 3: Figure S1A, Table 1), and that growth of the treatment-resistant lines was always significantly higher than that of the parental cell lines (Fig. 1a).

### EMT is associated with resistance to therapy

We used various methods to explore whether the cell lines effectively underwent EMT when acquiring resistance to therapy. First, we visually inspected the cells and assessed whether treatment-resistant cell lines displayed an elongated, spindle-like morphology with reduced cell-cell contacts as compared to their epithelial parental counterparts. This suggested that seven out of ten cell line pairs had undergone EMT (Fig. 1b and Additional file 4: Figure S2). We also profiled cell lines by RNA-sequencing and calculated for each cell line pair (treatment-resistant versus parental cell line) the log_2_ fold change (log_2_FC) in gene expression of epithelial and mesenchymal marker genes. Treatment-resistant cells upregulated various mesenchymal markers such as *VIM* and *CDH2*, whereas expression of epithelial markers, including *EPCAM* and *CDH1*, was downregulated (Fig. 1c). In line with this, EPCAM protein expression was reduced in nine out of ten treatment-resistant cell lines compared to parental cells (Fig. 1d, Additional file 5: Figure S3). In an effort to further explore these EMT-associated changes, we selected 50 genes reported to be involved in EMT (Additional file 1: Table S1). We quantified EMT based on the average differential gene expression of 16 epithelial and 34 mesenchymal genes in treatment-resistant versus parental cell lines (Fig. 1e). In seven out of ten treatment-resistant cell lines, expression of mesenchymal marker genes was upregulated relative to parental cell lines, while epithelial markers were downregulated (Fig. 1e and Additional file 3: Figure S1B for normalized read counts in individual cell lines). Therefore, out of the initial ten cell line pairs, we identify seven cell lines that underwent ‘full EMT’ and three that underwent only ‘partial EMT’ (Table 1). We will now focus on the seven cell lines undergoing full EMT, but will discuss the three cell lines undergoing partial EMT in a separate paragraph later in the manuscript.

### EMT is causing resistance to cancer therapy

To investigate whether EMT is either an epiphenomenon or causally underlying treatment resistance in the seven paired cell lines undergoing full EMT, we transfected both parental and treatment-resistant cell lines with three microRNAs of the microRNA 200 (miR-200) family, i.e., miR-200a, miR-200b, and miR-200c. These microRNAs are known to post-transcriptionally downregulate expression of various well-established mediators of the mesenchymal phenotype, such as *ZEB1*, *ZEB2*, *FN1*, *PPM1F*, *LOX*, and *MSN* [23–26]. To validate that transfection with miR-200 reverses treatment-resistant cell lines to an epithelial phenotype, we assessed EMT based on the expression of the 50-gene EMT signature (Additional file 1: Table S1). Upon miR-200 transfection, treatment-resistant cells exhibited reduced expression of mesenchymal markers relative to parental cells while gaining epithelial gene expression (Fig. 1f). When cells were transfected with a mock control, all cell line pairs retained their typical EMT expression (Additional file 3: Figure S1C).

To investigate whether EMT reversal also affects therapy resistance in these seven cell lines, we exposed mock and miR-200 transfected parental and treatment-resistant cells to the various cancer treatments at the parental IC_50_ concentration. Using sulforhodamine B colorimetric (SRB) assays, we again quantified cell growth. Growth after transfection with mock microRNA (miRNA) was nearly unaffected, with mock-transfected treatment-resistant cells still displaying a significant growth advantage over mock-transfected parental cells (Additional file 3: Figure S1D). However, miRNA-200 transfected treatment-resistant cells exhibited a substantially reduced growth when exposed to the treatment and even completely lost their original growth advantage compared to miRNA-200 transfected parental cells (Fig. 1g), confirming that EMT underlies resistance to therapy in these cell lines.

Although EMT is considered a reversible process occurring independently of genetic changes, it has been suggested to induce chromosomal instability [27]. The latter changes have been proposed as a principal underlying mechanism conferring therapeutic resistance to cancer [28]. For this reason, we investigated the extent to which treatment-resistant cells are genetically stable with respect to their parental counterparts, by performing low coverage whole genome sequencing (0.1–0.9-fold coverage, with the exception of HCC827P, 0.04-fold coverage) on all ten paired cell lines. This revealed that, with the exception of the 2 HepG2-derived cell line pairs, differences between parental and resistant cell lines were invariably small (Additional file 6: Figure S4). Overall, this confirms that EMT and not chromosomal instability underlies resistance to the treatment.

### Methylation of EMT gene promoters anti-correlates with EMT gene expression

To explore whether DNA methylation changes contribute to EMT-mediated treatment resistance, we used Illumina methylation beadchips to profile DNA methylation of parental and treatment-resistant cell lines. Some cell line pairs contained many cytosine guanine dinucleotides (CpGs) hypermethylated in treatment-resistant cell lines (defined as β_parental_ < 0.3 and β_resistant_ > 0.7), while others showed less distinct methylation changes (Additional file 7: Figure S5A).

**Fig. 2:**
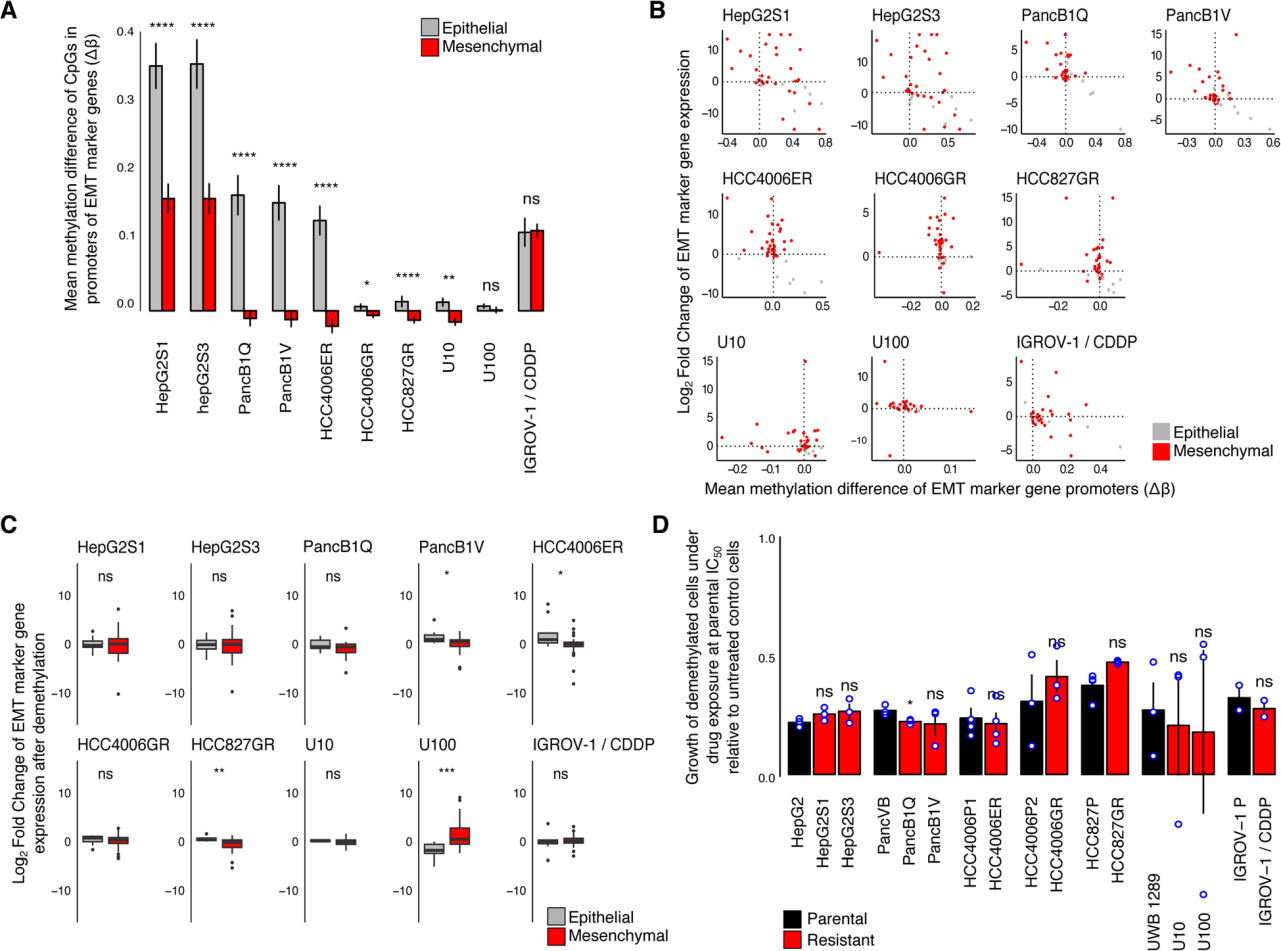
DNA methylation causally underlies EMT and resistance to therapy. **a** Methylation of EMT marker gene promoters. Shown are means ± SEM of the differences in DNA methylation (Δß values) between resistant and parental cells, of all CpGs profiled in the promoters of epithelial (117; gray) and mesenchymal (275; red) marker genes. Significance of the difference between mesenchymal and epithelial marker promoter methylation changes was calculated using a one-sided Mann-Whitney *U* test. **b** Correlation of EMT marker expression changes with promoter methylation changes. Shown are, per EMT marker gene, promoter methylation differences (difference in the average of CpGs, *x*-axis) versus differences in their expression (log_2_ fold-change, *y*-axis), between resistant and parental cells. Upon EMT, mesenchymal marker genes (red dots) are expected in the upper left quadrant and epithelial (gray dots) in the lower right quadrant. **c** EMT expression profile after pharmacological DNA demethylation using 5-aza-2′-deoxycytidine (0.5 μM, 8 days). Shown are differences in expression between resistant and parental cells of 16 epithelial (gray) and 34 mesenchymal (red) marker genes, as determined using RT-qPCR. Significance of the difference between epithelial and mesenchymal gene expression changes was calculated using a two-sided Mann-Whitney *U* test. **d** Growth of parental (black) and resistant (red) demethylated cells following exposure to the associated drugs at the parental cells’ estimated IC_50_ (Table 1)_,_ after an 8-day treatment with 0.5 μM 5-aza-2′-deoxycytidine, relative to control cells that were similarly demethylated but not exposed to the drugs. Shown are individual data points and means of experiments performed in triplicate (except for HCC4006P1 and HCC4006ER, HCC827P and HCC827GR, *n* = 4 and IGROV-1 and IGROV-1/CDDP, *n* = 2), each replication consisting of nine technical replicates. Significance of the difference between parental and resistant cell growth was calculated using a one-sided Mann-Whitney *U* test. *ns* non-significant, *p value < 0.05, **p value < 0.01, ***p value < 0.001, ****p value < 0.0001

However, when focusing on gene promoters of EMT markers, we observed more consistent changes across all seven paired cell lines. Specifically, promoters of epithelial marker genes were hypermethylated in all treatment-resistant versus parental cell lines, whereas promoters of mesenchymal marker genes were hypomethylated in five out of seven cell lines (Fig. 2a, Additional file 7: Figure S5B). In Fig. 2b, we summarize and correlate EMT-related promoter methylation changes with EMT marker expression changes (see Additional file 7: Figure S5C for data depicted per CpG). As expected, promoter methylation changes anti-correlate with gene expression changes. Particularly, in the seven treatment-resistant cell line pairs, mesenchymal marker CpGs were enriched (fraction of CpGs > 0.25) in the upper left quadrant (upregulation, hypomethylation) while epithelial marker CpGs were enriched in the lower right quadrant (downregulation, hypermethylation) (Additional file 7: Figure S5D and E).

### DNA methylation causally underlies EMT and resistance to treatment

To test whether DNA methylation changes in EMT gene promoters are not just correlative but also causally underlie EMT-driven treatment resistance, we exposed all cell lines to a non-cytotoxic dose of the passively demethylating agent 5-aza-2′-deoxycytidine (aza). Since 5-aza-2′-deoxycytidine is only effective during DNA replication [29], we cultured the cell lines under 5-aza-2′-deoxycytidine or vehicle (DMSO) treatment for at least 8 days (several cell doublings). We confirmed that following 5-aza-2′-deoxycytidine treatment, global DNA methylation levels were on average reduced by two-thirds of the original levels in all cell lines (Additional file 8: Figure S6A).

Should DNA methylation changes indeed underlie EMT-driven treatment resistance, we would expect expression changes of EMT markers in treatment-resistant versus parental cell lines to disappear, while growth under drug pressure of treatment-resistant cell lines exposed to 5-aza-2′-deoxycytidine would be significantly more reduced than that of parental cell lines. Indeed, after exposure to 5-aza-2′-deoxycytidine, the difference in EMT marker gene expression between parental and treatment-resistant cell lines had disappeared (Fig. 2c). When exposing the cell lines to vehicle, no such effects were observed (Additional file 8: Figure S6B). Additionally, after aza-induced demethylation, all treatment-resistant cells lost their growth advantage under therapeutic pressure compared to parental cells (Fig. 2d), which was not seen after exposure to vehicle (Additional file 8: Figure S6C).

Importantly, 5-aza-2′-deoxycytidine has previously been shown to induce off-target effects, such as DNA damage and cell cycle aberrations [30]. To exclude such confounders, we simultaneously inhibited all three DNA methyltransferases (DNMTs; *DNMT1*, *DNMT3A*, and *DNMT3B*) and explored the effect on EMT and therapy resistance in three cell line pairs (i.e., HepG2-HepG2S3, PancVB-PancB1V, and HCC4006P1-HCC4006ER). Upon transfection with a siRNA pool targeting *DNMT1*, *DNMT3A*, and *DNMT3B* (or pools of non-targeting siRNAs as a negative control), both mRNA and protein levels of DNMTs were significantly reduced (Additional file 9: Figure S7A-C). As expected, DNMT loss resulted in increased epithelial marker expression in all cell lines, and decreased mesenchymal marker expression in two out of three cell lines (Additional file 9: Figure S7D). Crucially, when assessing cell proliferation after DNMT knock down, resistant cells became more sensitive to the treatment (Additional file 9: Figure S7E), in line with the effects observed after 5-aza-2′-deoxycytidine-mediated DNA demethylation. Overall, these data indicate that DNA methylation causally underlies EMT-driven resistance to cancer treatment, rather than being a mere consequence.

### Characterization of DNA hydroxymethylation patterns

DNA methylation landscapes are shaped by the combined activities of the DNMTs and TETs [31]. We therefore explored *DNMT* and *TET* expression in RNA-seq data to show that DNMTs and TETs were abundantly expressed in each cell line (Additional file 10: Figure S8A, Additional file 11: Figure S9A). Similar observations were made by Western blot analysis for DNMT1 and DNMT3A. In most cell lines, we also noticed a small increase in DNMT expression when comparing resistant versus parental cell lines, although none of these differences reached statistical significance (Additional file 10: Figure S8B-C). Also, we failed to retrieve reliable antibodies to assess DNMT3B and TET expression.

**Fig. 3:**
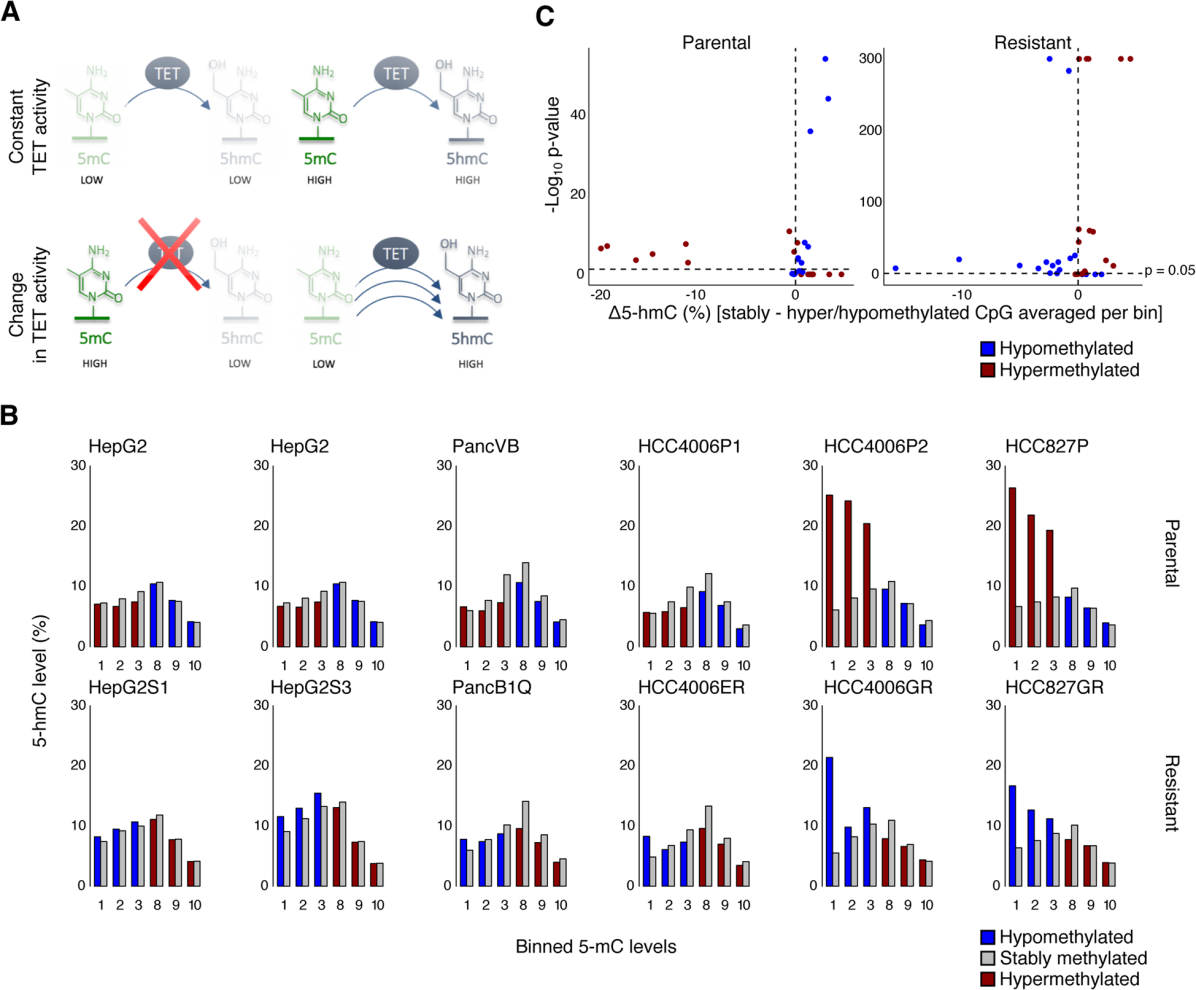
The role of TET activity in DNA methylation pattern changes. **a** Principle of TET-mediated differential methylation profiles. When TET-activity is constant, 5mC and 5hmC levels are expected to be correlated (upper panel). If a decrease in TET-activity is causing a CpG to be hypermethylated (lower left panel), a lower than expected 5hmC level is anticipated. If an increase in TET-activity causes CpGs hypomethylation (lower right panel), higher than expected 5hmC levels are anticipated. **b** Per cell line, hydroxymethylation of all CpGs included on the 450K array binned according to methylation levels, distributed over the *x*-axis. Bin 1 contains all CpGs with methylation levels between 0% and 10% methylation, bin 2 contains all CpGs with methylation levels between 10% and 20% methylation, etc. All bins containing both variable (hyper-/hypomethylated) CpGs as well as steady CpGs (no change in mC) are depicted. In each bin, averaged hydroxymethylation levels of hyper- or hypomethylated CpGs are depicted as red and blue bar plots respectively and compared to hydroxymethylation levels of CpGs with stable methylation levels, depicted as gray bar plots. Higher 5hmC levels for CpGs in the lower bins (1 to 3) and lower 5hmC levels in the upper bins (8 to 10) for variable CpGs relative to CpGs with steady 5mC levels, indicate that a difference in TET activity may underlie the differential methylation between parental and resistant cells. **c** For each bin depicted in (**b**), the difference in average 5hmC level of the stable CpGs minus the average 5hmC level of variable CpGs is plotted on the x-axis. On the *y*-axis, we depict the corresponding significance of the two-sided Mann-Whitney *U* test assessing whether the 5hmC levels of stably methylated CpGs differ significantly from the 5hmC levels of variably methylated CpGs (*p* value, -log_10_ scale)

To nevertheless characterize the role of DNMTs and TETs in establishing DNA methylation changes across different cell lines, we evaluated the dynamics of the epigenetic marks that these enzymes catalyze, i.e., 5-methylcytosine (5mC) and 5-hydroxymethylcytosine (5hmC), respectively. At CpGs where TET activity is stable, a constant turnover of 5-methylcytosine (5mC) into 5-hydroxymethylcytosine (5hmC) results in a stringent correlation between 5mC and 5hmC levels (Fig. 3a, upper panel). As such, at CpGs where TET activity is reduced, 5mC would increase and 5hmC would decrease (Fig. 3a, lower left panel), while vice versa, if TET activity is increased, 5hmC would increase and 5mC would decrease (Fig. 3a, lower right panel). With this in mind, we assessed 5hmC levels in CpGs that are differentially methylated between parental and treatment-resistant cells and compared these changes to 5hmC levels of CpGs with constant methylation levels. Particularly, we stratified CpGs from parental and resistant cell lines into ten bins (bin 1 containing CpGs with 5mC levels between 0 and 10%, bin 2 containing CpGs with methylation level between 10 and 20%, etc.). For each bin, 5hmC levels of CpGs that were differentially methylated between parental and treatment-resistant cells (red or blue) were compared to 5hmC levels of CpGs for which 5mC levels were not changed (gray). Since bins 4–7 only contain CpGs with relatively little changes in 5mC between treatment-resistant versus parental cell lines, these were not included in the analysis (Additional file 11: Figure S9B).

We observed that 5hmC levels for CpGs that became hypermethylated in treatment-resistant cell lines (going from bin 1–3 in parental cells to bin 7–10 in resistant cells, depicted in red in Fig. 3b, c) were high in parental cell lines (higher than constantly methylated CpGs in bin 1–3, depicted in gray), but decreased in treatment-resistant cell lines (lower than constantly methylated CpGs in bin 7–10, depicted in gray), suggesting that reduced TET activity at these sites underlies hypermethylation of these CpGs. Vice versa, 5hmC levels of CpGs that became hypomethylated in resistant cell lines (going from bin 7–10 in parental cells to bin 1–3 in resistant cells, depicted in blue in Fig. 3b, c) were characterized by slightly reduced 5hmC levels in parental cells (lower than constantly methylated CpGs in bin 7–10, depicted in gray) and by increased 5hmC levels in treatment-resistant cell lines (higher than constantly methylated CpGs in bin 1–3, depicted in gray), suggesting that increased TET activity underlies these differences. Interestingly, these differences (red versus gray, and blue versus gray) were significant (*P* < 0.05) in the majority (45/72) of the bins (Fig. 3c). We therefore propose that therapy resistance in these cell lines may at least partly be driven by TET-mediated changes in DNA methylation.

### DNA methylation mediates cross-resistance to cancer therapies

**Fig. 4:**
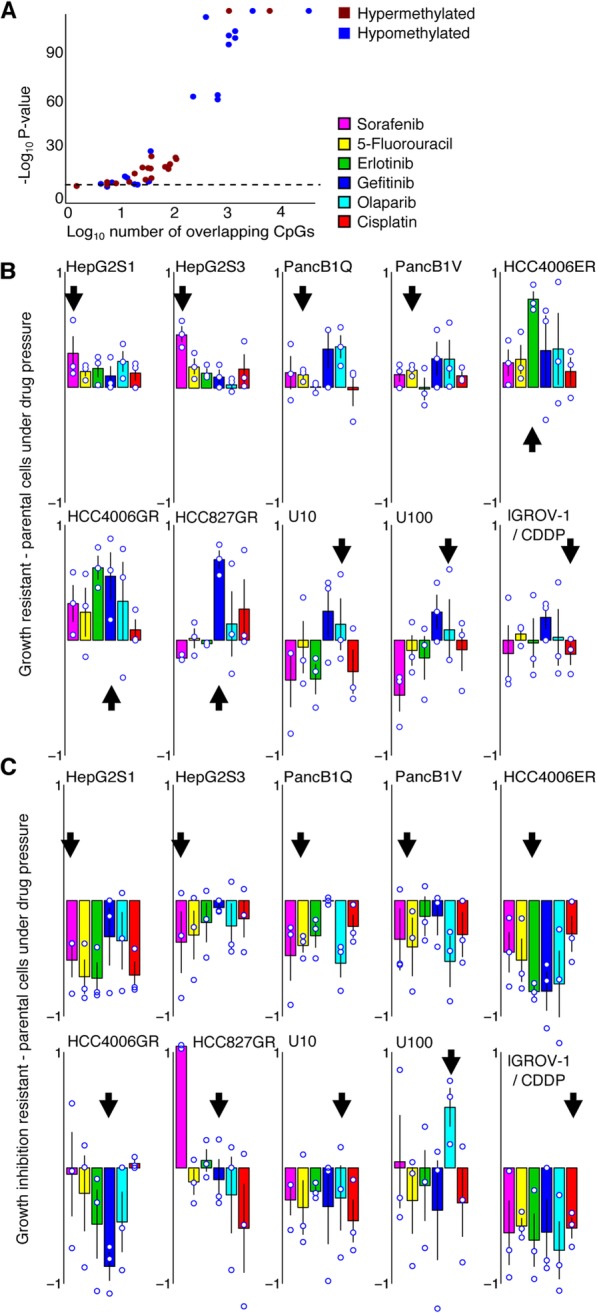
DNA methylation profiles that drive EMT are shared and confer cross-resistance. **a** The number of CpGs differentially methylated between resistant and parental cell lines, that are shared between pairs of resistant cell lines. Shown are the number of shared hyper- (red) and hypo- (blue) methylated CpGs for each resistant cell line pair (*x*-axis; log_10_ scale), versus the associated *p* values (*y*-axis; -log_10_ scale) of binomial tests assessing whether the number of shared hyper- or hypomethylated CpGs is significantly higher than expected. The horizontal dotted line indicates *p* value = 0.05. Primary data are shown in (Additional file 1: Tables S2, S3 and S4). **b** Cross-resistance of the resistant cell lines to various other therapies. Shown is the difference in growth between resistant and parental cells after exposure to the drugs indicated in the legend. Positive values indicate that resistant cells have a growth advantage upon drug exposure compared to their parental counterparts. Drug concentrations are as per (Additional file 1: Table S5). Cell growth was normalized to resistant and parental cells unexposed to drugs. Arrows indicate the drug to which the cells originally acquired resistance. Shown are individual data points and means of experiments that were performed in triplicate, each replication consisting of nine technical replicates. **c** Loss of cross-resistance after demethylation. As in panel (b), but for growth inhibition (as defined in ‘Methods’) of parental—resistant cells demethylated through an 8-day treatment with 0.5 μM 5-aza-2′-deoxycytidine. Negative values indicate resistant cell growth under drug pressure is more hindered after demethylation compared to their parental counterparts

Since we observed DNA methylation-driven EMT to underlie therapy resistance in several cancer cell lines and to various therapies, we assessed whether any of these differentially methylated CpGs were shared. We observed that the amount of differentially methylated CpGs shared between two cell lines was significantly higher than expected based on random chance (*P* < 0.05 for 38 out of 42 pairwise comparisons, Fig. 4a, Additional file 1: Tables S2, S3 and S4). This supports the notion of a common epigenetic mechanism, affecting a similar set of genes that underlies EMT-mediated resistance to therapy in these cell lines. We therefore explored whether there is also evidence of cross-resistance to other therapies. Specifically, we exposed all cell lines to five other drugs against which they had not been generated resistant. For this, we used IC_50_ concentrations of the corresponding parental cell line (Additional file 1: Table S5). Interestingly, the seven treatment-resistant cell lines exhibited extensive cross-resistance to other targeted therapies or chemotherapies (Fig. 4b). Moreover, cross-resistance was completely abrogated after demethylation of treatment-resistant cells with 5-aza-2′-deoxycytidine (Fig. 4c), further supporting our hypothesis that DNA methylation profiles causally underlie EMT-mediated resistance to various cancer therapies.

### Cell lines undergoing partial EMT show intermediate phenotypes

A subset of the treatment-resistant cell lines (i.e., U10, U100, and IGROV-1/CDDP) showed only intermediate or partial features of EMT. They did not develop a mesenchymal morphology upon resistance acquisition (Additional file 4: Figure S2) and, while *EPCAM* RNA and protein expression were reduced for two of the partial EMT cell lines (U10 and U100), the decrease was only subtle (Fig. 1c, Additional file 5: Figure S3). Overall, while mesenchymal marker gene expression was slightly upregulated, epithelial marker gene expression was not downregulated and the difference in expression change between epithelial and mesenchymal genes was not significant, in contrast to full EMT cell line pairs (Fig. 1e). At the DNA methylation level, only one partial EMT model showed changes consistent with EMT (U10), while in two of three partial EMT models, CpGs in the promoters of both epithelial and mesenchymal marker genes were hypermethylated (Fig. 2a, Additional file 7: Figure S5B). As mentioned, promoter methylation anti-correlated with gene expression (Fig. 2b, Additional file 7: Figure S5C). Accordingly, enrichment of mesenchymal marker CpGs in the upper left quadrant (upregulation, hypomethylation) and of epithelial marker CpGs in the lower right quadrant (downregulation, hypermethylation) was less pronounced (or absent) in cell line pairs undergoing partial EMT than what was seen for the full EMT models (Additional file 7: Figure S5D and E). Partial EMT has been suggested to be important in cancer dissemination [32]. Its role in resistance acquisition has not yet been assessed and a detailed study is beyond the scope of our report. Nonetheless, reduction of resistance after pre-treatment with the demethylating agent 5-aza-2′-deoxycytidine was also seen after pre-treatment with vehicle (Fig. 2d, Additional file 8: Figure S6C). However, as opposed to the full EMT models, these partial EMT models did not show extensive cross-resistance (Fig. 4b).

### DNA methylation-driven EMT underlies resistance to therapy in HCC patients

To explore the clinical relevance of our in vitro findings, we assessed whether DNA methylation-driven EMT also represents a mechanism of acquired resistance to targeted therapies in cancer patients. To this end, we developed a methylation assay that is able to accurately quantify DNA methylation levels in low-input circulating cell-free DNA (cfDNA), and more specifically, monitor methylation changes in EMT genes (see ‘Methods’ section for a detailed description). Briefly, after bisulfite conversion of cfDNA, a specific library preparation protocol was developed to process the heavily fragmented DNA. Next, a subset of the genome was captured by a pool of capture probes using the SeqCap Epi Choice S Enrichment Assay. In order to enhance specificity for ctDNA, this pool was designed to specifically target CpGs that are lowly methylated in the blood of healthy individuals. Particularly, a published 450K methylation dataset on blood samples of 656 healthy subjects was used to select 47,569 CpGs with an average β value < 0.03 across all subjects [33], resulting in a design of 28,025 probes with a median size of 149 base pairs (range 59–1037 base pairs).

**Fig. 5:**
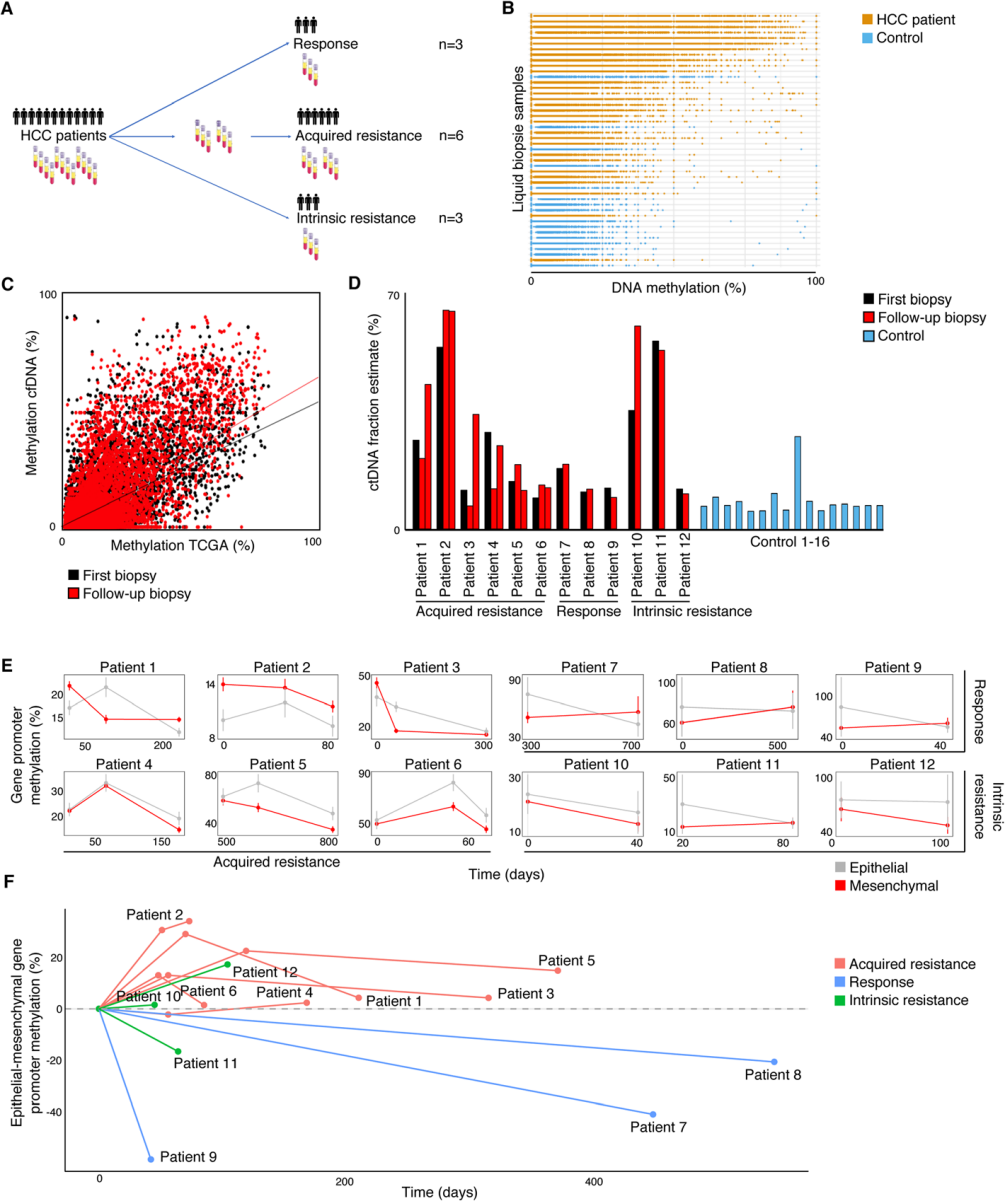
cfDNA Methylation profiles in HCC patients match our in vitro findings. **a** Study set-up. For each patient, a sample at treatment onset was collected. For the three intrinsically resistant patients, a second sample was taken at first follow-up, corresponding to the end of treatment. For the six patients with acquired resistance, a second sample was taken under treatment, while still responsive, and a third upon resistance acquisition, corresponding to the end of treatment. For the three patients showing durable response, a second sample was collected at the end of the study. **b** Boxplots depict methylation levels of target CpGs in samples from 16 control subjects (blue) and 12 HCC patients (yellow). Samples are ordered according to mean target CpG methylation. **c** Correlation between the target CpG methylation levels in HCC patients (TCGA, *n* = 370; mean depicted on the *x*-axis) and corresponding levels in first (black) and last (red) liquid biopsies of Patient 1 (*y*-axis). Correlation direction coefficients depicted by the straight lines indicate cfDNA fractions estimated to originate from tumor tissue. See ‘Methods’ for details. **d** Bar plots depict the estimated fraction ctDNA in each sample for HCC patients (left panel, start samples in black and follow-up samples in red) and control samples (right panel, blue). **e** Evolution in time (days under treatment; *x*-axis) of estimated methylation levels of epithelial (gray) and mesenchymal (red) gene promoter CpGs (*y*-axis). Shown are means ± SEM. Panels in left half show data for acquired resistance patients, on the upper right for responding patients, and on the lower right for intrinsically resistant patients. **f** Evolution in time (days under treatment; *x*-axis) of mean epithelial minus mean mesenchymal gene promoter methylation (*y*-axis), relative to the *t*_0_ (time point 0) situation. Shown are data from patients with acquired resistant (red), responding patients (blue), and intrinsically resistant patients (green)

Next, we applied this novel method to blood samples collected from healthy individuals (*n* = 16) and to serially collected blood samples from advanced HCC patients treated with first-line sorafenib (*n* = 12, Additional file 1: Table S6). For each patient, we collected a blood sample at treatment onset. Three patients showed clear progressive disease at first evaluation and at that time, a second blood sample was obtained (i.e., intrinsic resistance). In six patients, disease control (i.e., partial remission or stable disease) was initially seen but resistance developed later on (i.e., acquired resistance). In this cohort, we collected an on-treatment sample as well as a sample at time of progressive disease. Three patients were clearly responding at time of last sample collection (i.e., response). This includes one patient treated with sorafenib for > 2 years, stopped after myocardial infarction (patient 7), and one patient still receiving sorafenib at time of writing the manuscript (almost 3 years treatment, patient 8) (Fig. 5a, Additional file 1: Table S6).

We reasoned that methylation levels measured in plasma (*M*_meas_) represent the sum of methylation levels measured in ctDNA (*M*_tumor_) and other cfDNA sources (*M*_cfDNA_). Therefore, *M*_meas_ can be modeled as *M*_meas_ = *M*_tumor_ * f_ctDNA_ + *M*_cfDNA_ * f_cfDNA_, with f_ctDNA_ being the fraction of ctDNA and f_cfDNA_ the fraction of other sources of cfDNA (with f_ctDNA_ + f_cfDNA_ = 1). Given the initial selection procedure of target CpGs, we expect *M*_cfDNA_ to be near 0; hence, we can approach *M*_tumor_ by *M*_meas_/f_ctDNA_. First, we assessed whether the target CpGs exhibit the expected low cfDNA methylation levels in the 16 samples collected from healthy individuals (*M*_cfDNA_ ≅ 0; Fig. 5b). Target CpGs in healthy individuals indeed showed markedly lower methylation levels compared to blood samples from HCC patients. Additionally, methylation of these target CpGs was significantly lower than methylation of other CpGs included on the probes that are not target CpGs (Additional file 12: Figure S10A). CpG methylation levels from the healthy individual samples moreover correlated strongly with methylation levels extracted from an independent WGBS dataset of a normal blood sample [34], further validating our experimental design (Additional file 12: Figure S10B and C). Lastly, to confirm that our design specifically detects ctDNA methylation, and not cfDNA derived from other sources in the blood, we correlated DNA methylation levels from blood to those obtained from a corresponding tumor biopsy. Particularly, for one patient (i.e., patient 6), both a tissue biopsy and cfDNA were available. We observed that the correlation between the tissue biopsy and cfDNA data from this patient was more significant and stronger than for all other correlations, suggesting that our assay specifically measures patient-specific tumor DNA methylation profiles from blood (Additional file 12: Figure S10D, Additional file 1: Tables S7 and S8).

To assess EMT in the HCC patients using our novel assay, we first evaluated EMT marker promoter methylation in the blood samples from healthy individuals (Additional file 12: Figure S10E and F). In order to faithfully approach *M*_tumor_ by *M*_meas_/f_ctDNA_ (which is only true when M_cfDNA_ ≅ 0), we excluded 13 EMT markers (see ‘Methods’ for details) from further analysis based on variable or high methylation levels in the cfDNA from healthy individuals. Next, we estimated f_ctDNA_ in each sample as follows: with f_ctDNA_ approximating *M*_meas_/*M*_tumor_ if M_cfDNA_ ≅ 0, we calculated f_ctNDA_ as the increment of the correlation *M*_meas_ versus *M*_tumor_ for all target CpGs. *M*_tumor_ values were estimated using average methylation values of the 370 HCC patients for whom 450K methylation data is available in The Cancer Genome Atlas (TCGA). An example of this analysis is given in Fig. 5c. As expected, f_ctDNA_ estimates for most patient samples were significantly higher than for control samples (Fig. 5d).

Finally, we assessed methylation changes of nine epithelial and 28 mesenchymal gene promoters in each of the patients by plotting their average estimated methylation levels each time a blood sample was assessed (Fig. 5e). In all six patients with acquired resistance, mesenchymal promoters were, for instance, severely hypomethylated in time, suggesting the upregulation of mesenchymal genes and acquisition of EMT to underlie resistance to sorafenib. However, since EMT is determined by both epithelial to mesenchymal gene expression, we also calculated methylation changes in epithelial genes relative to those in mesenchymal genes levels in all blood samples from all patients (Fig. 5f). In the six patients with acquired resistance, promoters of epithelial genes gradually gained methylation relative to mesenchymal gene promoters, suggesting these tumors had undergone EMT. In the three intrinsically resistant patients, no specific trend was noticed, with patient 12 developing hypomethylation of mesenchymal gene promoters and patient 11 showing the opposite. Finally, blood samples taken from responding HCC patients showed a more epithelial methylation profile over time, confirming the observation that EMT can underlie resistance to sorafenib treatment in HCC patients.

Overall, these data provide in-patient support for our in vitro observation that methylation-driven EMT can underlie acquired resistance to multiple cancer therapies.

## Discussion

The extent to which EMT underlies metastasis and resistance to cancer therapy has been the subject of a long-standing debate [1, 3, 35, 36]. Its transient and reversible nature, the existence of several intermediate stages of EMT, the involvement of numerous biological pathways, and the vast number of factors favoring an epithelial or mesenchymal cell state render EMT a complex and difficult to study phenomenon [5]. As a result, there are many outstanding questions regarding EMT. For instance, it is not clearly established which cancer types more readily undergo EMT, which cancer therapies more likely induce EMT-mediated resistance, and, most importantly, how frequently EMT is involved in tumors developing therapy resistance in cancer patients [1, 5, 35].

Here, we established that in spite of their developmental and (epi-)genetic differences, several cell lines derived from various cancer types, including HCC, pancreatic, lung and ovarian cancer, acquired resistance to cancer therapies by activating an EMT program. Moreover, we found that upon developing resistance, these cell lines—although they displayed widely varying methylation dynamics—all acquired similar methylation changes at the promoters of 50 pre-defined EMT marker genes. Particularly, epithelial gene promoters became significantly more methylated, while mesenchymal gene promoters adopted a less methylated state. Various mechanisms could contribute to these methylation changes. Indeed, we showed that expression levels of the main DNA methylation modifying enzymes (*DNMTs* and *TETs*) are high, both in parental and resistant cell lines, suggesting that any of these enzymes could contribute to the observed effects. At least for DNMT1 and DNMT3A, we failed however to observe prominent changes in expression comparing resistant versus parental cell lines, suggesting that at least both enzymes are not involved in mediating the resistance phenotype. However, also a redistribution in any of the DNMTs and/or TETs at specific genomic locations, or even a transient change in expression of DNMTs or TETs in specific genomic regions while acquiring treatment resistance could contribute to the observed phenotype. For instance, transient increases in DNMT and TET localization, respectively at epithelial and mesenchymal gene promoters could drive the methylation changes. A limitation of our study is therefore that cells collected at multiple time points during resistance acquisition were not available, and that we therefore did not perform ChIP-Seq for any of the DNMTs or TETs to assess their genomic location during resistance acquisition. Nevertheless, our observed differences in 5mC and 5hmC in parental versus resistant cells suggest that the epigenetic alterations in resistant cells are at least partly mediated by changes in DNA demethylation activity. In line with this, TET activity at different loci has indeed been linked both to induction and inhibition of EMT [37, 38].

Furthermore, we established causality of these methylation changes in the acquisition of resistance to cancer therapy via an EMT-mediated mechanism. This was demonstrated by exposing treatment-resistant cell lines during several cell divisions to the demethylating agent 5-aza-2′-deoxycytidine, thereby reversing EMT and resistance. Importantly, *DNMT* knock down recapitulated these findings, thus excluding that the effects of 5-aza-2′-deoxycytidine treatment are attributable to off-target effects such as DNA damage or cell cycle aberrations. Notably, methylation-driven EMT was observed in cell lines resistant to a wide variety of chemotherapies, including the widely-used 5-fluorouracil and cisplatin-based chemotherapies, as well as several targeted agents, including sorafenib, gefitinib, erlotinib, and olaparib. Overall, this underscores the role of DNA methylation in maintaining a stable mesenchymal or epithelial cell state, and in mediating the response to various cancer therapies. Furthermore, our data suggest that manipulating DNA methylation (either genome-wide or via a more targeted approach) could represent a viable strategy to overcome EMT-mediated acquired resistance to a variety of cancer therapies.

Importantly, we also demonstrate that DNA methylation-driven EMT can underlie treatment resistance to first-line sorafenib treatment in patients with advanced HCC. Particularly, acquired resistance in initially responsive HCC patients was always accompanied by methylation changes indicative of EMT, whereas patients still responding to the therapy consistently failed to undergo such changes. Unfortunately, it was difficult to also assess whether patients intrinsically resistant to the treatment failed to respond because they already exhibited a mesenchymal phenotype. As such, intrinsic resistance to sorafenib is possibly caused by mechanisms other than DNA methylation-driven EMT. Nevertheless, our data demonstrate that EMT-driven DNA methylation is also in patients a frequent mechanism underlying acquired resistance to cancer therapy. Assessing the DNA methylation status of EMT gene promoters in liquid biopsies should therefore be considered a potential biomarker of response to sorafenib in HCC.

Finally, we also developed a novel method for targeted DNA methylome profiling of liquid biopsies. To reduce amplification bias and improve library depth and representation, we first subjected the cfDNA to bisulfite conversion and only then ligated the fragmented and C/T-converted DNA with library preparation adapters. This allowed us to generate high-quality libraries using minimal cfDNA input. We developed a capture panel based on the SeqCap Epi technology that specifically captures 47,569 CpGs that are unmethylated in the blood of 656 healthy individuals [33], allowing us to interrogate the methylation status of CpGs in 37 EMT gene promoters that are unmethylated in healthy subjects, but possibly methylated in cancer patients depending on the EMT status of the corresponding tumor. Indeed, when correlating DNA methylation profiles measured in 12 HCC patients to DNA methylation levels obtained from pure tumor tissue from one patient, correlations were strongest between blood and tissue samples obtained from the same patient. The fact that we can track EMT in serial liquid biopsies using DNA methylation profiling opens many different possibilities. First of all, this minimally invasive approach may be leveraged to similarly monitor dynamic changes in EMT patients with other cancer types, treated with other therapies. Importantly, it also illustrates that other dynamic cancer cell phenotypes can be monitored in blood.

## Conclusions

Herein, we show that DNA methylation changes underlie EMT in various cancer cell lines as a common mechanism of resistance to both targeted and chemotherapy-based treatments. We provide the first evidence that EMT can be monitored in blood by assessing the DNA methylation status of CpG sites in EMT genes and can be used as a predictive marker of resistance to sorafenib. These findings not only illustrate how these EMT-associated epigenetic changes are clinically relevant as a potential biomarker but also provide proof-of-principle on how dynamic changes of cancer cell phenotypes can be non-invasively monitored in blood.

## Methods

### Materials

All materials were molecular biology grade. Unless explicitly stated, all materials were from Sigma.

### Cell lines

All information about the origin, generation, and references of all cell lines is summarized in Table 1. HepG2 cell lines were cultured in William’s E medium with 10% fetal bovine serum (FBS) and 1% penicillin-streptomycin (PS). All other cells were cultured in RPMI 1640 glutaMAX medium with 10% FBS and 1% PS. Cells were cultured at 37 °C, in atmospheric oxygen concentration (21%) with 5% CO_2_. Splitting of cells was performed using trypsin-EDTA. Cell cultures were regularly confirmed to be mycoplasma-free via PCR detection. In general, cells were used for experiments after one to three passages.

### Drugs

All drugs were solubilized in 10% DMSO. All drugs were purchased from Sigma except for olaparib (Bioconnect) and sorafenib (Enzo Lifesciences).

### Resistance assessment

To determine IC_50_ values, dose response curves were generated using the Sulforhodamine B colorimetric assay [22]. To this end, 5000 cells were seeded per well in 3 × 12 wells of a flat-bottomed 96-well plate. Parental and resistant cells were seeded on the same plate to avoid batch effects. In a separate plate, 5000 cells/well were seeded in 6-wells per cell line. After 24 h, this plate was fixed using trichloroacetic acid for 1 h at 4 °C. In the other plate, both parental and resistant cells in well 1 to 9 were exposed to the drugs they were made resistant to. From well 1 to 9, the concentration of the drugs followed a twofold dilution, with the concentration in well 5 being the cell line specific IC_50_ for this drug as was found in literature. Well 10 to 12 served as controls and were treated with 10% DMSO. After a 72-h incubation, the plates were fixed, stained, and analyzed following manufacturer’s instructions. Growth was calculated as described [22].

For resistance assessments after manipulation of the cells (e.g., after reversal of EMT, pharmacological demethylation, DNMT knock down, or the respective vehicle treatments), control cells were seeded in wells 10 to 12 (exposed to 10% DMSO) and cells in wells 1 to 9 were exposed to drugs (solubilized in 10% DSMO) at the parental IC_50_ as was determined by the dose-response curves. The standard protocol was followed and growth after drug exposure was calculated as described in the SRB protocol. Growth inhibition was defined as the difference in growth between vehicle-treated (mock miRNA/DMSO/mock siRNA) and treated (miR-200/aza/siRNA DNMT1/3A/3B) cells.

### DNA extraction

Cultured cells were washed with DPBS and detached with trypsin-EDTA (0.25%) (Thermofisher). Cell pellets were obtained by centrifugation and were washed once in DPBS. Subsequently, nucleic acids were extracted using the Wizard Genomic DNA Purification kit (Promega) according to manufacturer’s instructions. All buffers were supplemented with deferoxamine (DFO, 200 μM). DNA was dissolved in 80 μL PBS-DFO with RNaseA (200 U, NEB) and incubated for 10 min at 37 °C. Next, proteinase K (200 U) was added and the mixture was incubated for 30 min at 56 °C. DNA was purified using the QIAQuick blood and tissue kit (all buffers supplemented with DFO). Elution was performed in 100 μL of a 10 mM Tris, 1 mM EDTA solution (pH 8) and stored at − 20 °C until further processing.

### Low coverage whole genome sequencing

Shot-gun whole genome libraries were prepared using the KAPA library preparation kit (KAPA Biosystems). Whole genome DNA libraries were created, according to manufacturer’s instructions. Before end-repair, a 4-h incubation step at 65 °C was added to remove as many reversible crosslinks as possible, after which excessive single stranded DNA was removed using Mung-Bean nuclease. The concentration of double-stranded DNA was reassessed using Pico-green and the concentration of adapters used in the ligation step of the library construction was modified based on the DNA measured. For the library enrichment, 5–15 cycles of PCR with intermediate assessment steps were used instead to ensure low adapter dimer content and high library yield. Following quantification with qPCR, the resulting libraries were sequenced on a HiSeq2500 or HiSeq4000 (Illumina) at low coverage (± 1×) for shallow-seq. Raw sequencing reads were mapped to the human reference genome (NCBI37/hg19) using Burrows-Wheeler Aligner (BWA v0.5.8a). Picard (v1.43) was used to remove PCR duplicates. Somatic copy number aberrations (CNAs) were identified by binning the reads in 30 Kb windows, correcting for genomic waves using the PennCNV software package and the resulting number of reads per 30 Kb window were transformed into log *R* values. The ASCAT algorithm version 2.0.1 was used to segment the raw data and estimate ploidy.

### RNA extraction and cDNA synthesis for qPCR

Cell culture medium was removed, cells were washed with DPBS, TRIzol (Life Technologies) was added, and cells were processed according to manufacturer’s guidelines. Reverse transcription was carried out using SuperScript II Reverse Transcriptase according to manufacturer’s guidelines.

### RNA sequencing

RNA was extracted as described above. Libraries were prepared using either the Stranded mRNA-Seq Kit (KAPA) or the QuantSeq 3′mRNA-Seq Library Prep kit (Lexogen) KAPA. Up to 4 μg of total RNA was used as an input for the Stranded mRNA-Seq Kit (KAPA). mRNA is captured using magnetic oligo-dT beads: while mRNA is retained, other RNA is washed away in two subsequent washes. mRNA fragments are fragmented and converted to cDNA, with dUTP incorporated in to the second cDNA strands. A-tailing, adapter ligation, and enrichment are carried out to obtain libraries. Enrichment of the libraries is achieved by using KAPA HiFi Hotstart Readymix. The strand marked with dUTP is not amplified, allowing strand specificity. Libraries were sequenced single end, on an Illumina HiSeq 2500. Adaptors were removed using FastXClipper and reads were mapped using Bowtie and TopHat. Counts were extracted using HTSeq after sorting the mapped files using SAMtools.

### Lexogen

Around 500 ng total RNA was used as an input for QuantSeq 3′ mRNA-Seq Library Prep (Lexogen). The double-stranded cDNA library was made by first-strand synthesis with an oligo-dT primer containing the Illumina-specific Read 2 linker and second-strand synthesis with random primer containing the Illumina-specific Read 1 linker sequence. Library amplification introduced a unique i7 index for each sample and sequences required for cluster generation. Libraries were sequenced single end, on an Illumina HiSeq4000. Optical duplicates were removed using clumpify. Adaptors were removed using FastXClipper and reads were mapped using Star. Counts were extracted using HTSeq after sorting the mapped files using SAMtools.

Gene expression counts were normalized to total read count and expressed in counts per million (CPM). Log_2_ fold change (Log_2_FC) values per gene were calculated from these values, where Log_2_FC > 0 indicates upregulation in the resistant cells and Log_2_FC < 0 indicates downregulation.

### EPCAM protein expression profiling

Cells were collected by trypsinization. Cell pellets were solubilized in 500 μL DPBS + 4% FBS and subsequently diluted to 10^6^ cells per mL. Antibody (5 μL of stock solution) or eFluor450 (5 μL of 200 μg/mL stock solution) was added to 1 mL of 10^6^ filtered cells in FACS tubes. Cells with antibodies were incubated on ice for 30 min before analyzing them on a BD FACSVerse Flow Cytometer. Results were analyzed using the FlowJo software: first cells were gated to exclude signal coming from cell debris and doublets. Subsequently, gating was done to obtain results for live cells only. Ultimately, forward scatter area (FSC-A) versus EPCAM antibody (APC-conjugated) signal was displayed.

### Western blot

Protein was extracted from cells at 80–90% confluency in 6-well plates or 10 cm dishes. RIPA buffer with freshly added protease inhibitor was added to the wells; the lysed cells were collected using cell scrapers and transferred into Eppendorf tubes. After 30-min incubation on ice, the samples were pulled through a 1 mL needle. To remove cell debris, samples were centrifugated for 5 min at 10,000 rpm; the supernatant was transferred into a fresh Eppendorf. Protein concentration was determined using the Pierce BCA protein assay kit (Thermofisher) according to manufacturer’s instructions. Then, 50 μg of protein was mixed with 5 μL loading dye and 2 μL of 10× sample reducing agent in a total volume of 20 μL RIPA and boiled for 5 min. Samples were loaded onto NuPAGE™ 4–12% Bis-Tris Protein Gels (Invitrogen) together with a reference marker, consisting of 10 μL of MagicMark XP Western Protein Standard (Thermofisher) and 10 μL Precision Plus Protein Kaleidoscope Standard (Bio-Rad). The Trans-Blot Turbo Transfer System (Bio-Rad) was used for transfer of the protein from gel to membrane (both PVDF and Nitrocellulose were used) according to manufacturer’s instructions. The membranes were incubated in 5% non-fat milk at room temperature for 2 h for blocking after which primary antibody was added at the concentration recommended by the manufacturer. For DNMT1, we used NB100-56519 (NovusBio). For DNMT3A, we used 2160S (Cell Signaling). Membranes incubated with primary antibodies were incubated overnight at 4 °C. The following morning, membranes were washed four times in PBS-tween0.05% during 1–2 h (at room temperature) after which they were incubated with HRP-conjugated secondary IgG antibody (7074S and 7076S, depending on the primary antibody, Cell Signaling) in 5% milk for 2 h at room temperature. Before imaging on a Licor device, the membranes were washed three times in PBS-tween0.05% during 2 h. The Novex™ ECL Chemiluminescent Substrate Reagent Kit (Thermofisher) was used for detection of highly expressed proteins and the SuperSignal™ West Femto Maximum Sensitivity Substrate kit (Thermofisher) for lowly expressed proteins. For protein expression quantification, Fiji (ImageJ) software was used.

### OpenArray

RNA was extracted as described above and concentration was adjusted to 2 μg in 10 μL. Reverse transcription was performed using High Capacity cDNA Reverse Transcription kit (Life Technologies). cDNA is diluted 5-fold before enriching the targets for gene expression analysis with Taqman PreAMP Master Mix Kit (10 cycles). The enriched cDNA targets were 5-fold diluted before mixing with the TaqMan Open Array Mastermix, after which the mix was loaded on the OpenArray slides (Thermofisher). Cycle threshold (*C*_t_) values were determined for each cell line, group (parental/resistant), condition (treated/control), and gene (50 EMT genes + five housekeeping genes) contained on the array in two technical duplicates and normalized according to the corresponding amplification efficiency. For each cell line, we determined the two out of the five included housekeeping genes (*ACTB*, *B2M*, *GAPDH*, *UBC*, *YWHAZ*) that were most stably expressed across both groups and all conditions to use for normalization. Log_2_FC from parental to resistant samples per gene were calculated from the normalized *C*_t_ values averaged over the two technical duplicates using the delta-delta *C*_t_ method.

### miRNA experiments

To reverse EMT, we transfected the cells in 6-well plates (Elscolab) (125,000 cells diluted in 2 mL of medium) with either three miRNAs from the miRNA-200 family (hsa-miR-200a-3p, hsa-miR-200b-3p, and hsa-miR-200c-3p) or with a negative control miRNA (all Thermofisher Scientific). First, 5 μL of RNAiMAX Liptofectamine (Thermofisher Scientific) in 250 μL in Opti-MEM® I reduced serum medium (Thermofisher Scientific) was added to each well containing 30 pmol of each miRNA in 250 μL Opti-MEM. After 15-min incubation at room temperature, the diluted cells were added dropwise and mixed gently. After 24–72 h incubation at 37 °C, the cells were evaluated for EMT expression (OpenArray), morphology (visually), and drug resistance (SRB assay).

### CpG specific (hydroxy) methylation assessment

5mC and 5hmC values were obtained using Illumina’s Infinium HumanMethylation450 (‘450 K’) and Infinium HumanMethylationEPIC BeadChips (‘EPIC’). To obtain 5hmC values through TET-assisted bisulfite chromatin immunoprecipitation (TAB-ChIP), extracted DNA was glycosylated and oxidized using the 5hmC TET-assisted bisulfite sequencing (TAB-Seq) Kit (WiseGene) [39] and subsequently processed using the manufacturer’s standard protocol as for 5mC values. The samples were subjected to bisulfite conversion, DNA amplification, and array hybridization. Data processing was done using Bioconductor’s minfi package in R. Briefly, raw intensity files were read into R. Samples were normalized using Subset-quantile within array normalization (SWAN). To obtain 5mC specific ß values, TAB-ChIP generated normalized ß values were subtracted from standard 450 K generated normalized ß values.

### Global (hydroxy) methylation levels

Global (hydroxy) methylation levels were obtained through liquid chromatography/mass spectrometry (LC/MS). Cells were washed with DPBS and collected as a cell pellet in a microcentrifuge tube. Cells were lysed using 750 μL of nuclei lysis buffer (Promega) and incubated at 65 °C for 20 min. Proteins were precipitated using protein precipitation buffer (Promega) and centrifugation. The supernatant was mixed with an equal volume isopropanol, to precipitate the DNA. The DNA pellet was dissolved in DPBS and treated with RNAse A solution, Proteinase K, and AL buffer (Qiagen). After an incubation step at 56 °C, the sample was mixed with ethanol, loaded on to a DNeasy mini spin column (Qiagen), washed, and eluted according to the manufacturer’s protocol.

To determine global 5mC and 5hmC DNA levels before and after 5-aza-2′-deoxycytidine treatment, 1 μg DNA in 50 μL H_2_O was digested in an aqueous solution (7.5 μL) of 480 μM ZnSO_4_, containing 42 U nuclease S1, 5 U Antarctic phosphatase, and specific amounts of labeled internal standard were added and the mixture was incubated at 37 °C for 3 h in a Thermomixer comfort (Eppendorf). The resulting cytosine, 5mC and 5hmC peaks, were normalized using the isotopically labeled standards and expressed relative to the total cytosine content (C + 5mC + 5hmC).

### Demethylation experiments

To demethylate the cells, they were incubated with 0.5 μM of the passively demethylating agent 5-aza-2′-deoxycytidine (aza, Sigma Aldrich). Since 5-aza-2′-deoxycytidine is a cytotoxic compound, we first determined an appropriate dose by establishing dose-response curves. None of the cells’ viability was affected after exposure to 5-aza-2′-deoxycytidine at 0.5 μM, while DNA methylation levels were satisfactory reduced in all model cell lines. At higher doses, cell viability was affected while demethylation rates did not increase concordantly. For demethylation experiments, medium containing 5-aza-2′-deoxycytidine was refreshed every other day. Cells were incubated with 5-aza-2′-deoxycytidine for at least three cell doublings (8 days) at 37 °C, after which the cells were evaluated for global methylation levels (LC/MS), morphology (visual), EMT expression (OpenArray), and resistance to various therapies (SRB assay).

### DNMT knock down experiments

To inhibit the DNA methyltransferases, we transfected the cells in 6-well plates (Elscolab) (125,000 cells diluted in 2 mL of medium) with siRNAs targeting *DNMT1*, *DNMT3A*, and *DNMT3B* or with non-targeting control siRNAs (all HorizonDiscovery/Dharmacon siRNA SMARTpools). First, 5 μL of RNAiMAX Liptofectamine (Thermofisher Scientific) in 250 μL in Opti-MEM® I reduced serum medium (Thermofisher Scientific) was added to each well containing 40 nmol of each siRNA in 250 μL Opti-MEM. After 15-min incubation at room temperature, the diluted cells were added dropwise and mixed gently. After a 24-incubation at 37 °C, the cells were transfected again under the same conditions. Another 48 h later, they were evaluated for DNMT RNA and protein levels (qPCR and Western Blot). After 5 days, cells were evaluated for EMT expression (RNA-seq) and drug resistance (SRB assay).

### Liquid biopsy samples

Blood samples were collected in EDTA tubes from patients with advanced HCC after written informed consent. Samples were taken before starting or at the start of sorafenib treatment, at time of follow-up imaging and at the emergence of resistance (if applicable).

cfDNA extraction of liquid biopsy samples. Samples were processed within 2 h after collection from the patients using QIAGEN’S DNeasy Blood and Tissue kit according to manufacturer’s instructions.

### Target MethylSeq of cfDNA

In order to enhance specificity for ctDNA, which commonly constitutes only a small fraction of the cfDNA [40], we set up a targeted bisulfite sequencing approach interrogating only CpGs (*n* = 47,569) that are characterized by low methylation levels (β_avg_ < 0.03) in the blood of healthy patients based on the publicly available dataset GSE40279 from Hannum et al. [33]. Bisulfite conversion was performed using the EZ DNA Methylation-Lightning Kit (D5031-200 reactions) from Zymo Research, according to manufacturer’s instructions with minor adjustments: input concentration per sample was 10 ng cfDNA and final elution was performed in 15 μL instead of the prescribed 20 μL. Library preparation was performed using the Swift Accel-NGS 1S Plus DNA library kit (WB10096, 96 reactions) from Westburg, using a slightly adapted protocol. The adaptase reaction was performed at 75% of the recommended reagent volume; 4.875 μL Low EDTA TE was used to compensate for the surplus of input sample volume used (total volume of 30 μL instead of the prescribed 40 μL). The extension reaction was performed using 75% of the recommended reagent volume of each reagent (total volume of 65.25 μL instead of the prescribed 87 μL), except for buffer W3 and enzyme W4 that were replaced with equivalent volumes of KAPA HiFi Hotstart Uracil+. Pre-ligation, we performed a 1.2× Ampure XP bead clean up, eluting in 20 μL. Post-ligation, we performed a 1.2× Ampure XP bead clean up, eluting in 16 μL. Indexing PCR reaction was run adding 4 μL of reagent W2, 25 μL KAPA HiFi Hotstart Uracil+, and 5 μL index (16024) or 2 × 2.5 μL (18096), using KAPA PCR cycling settings for 12 PCR cycles. Post-PCR, we performed a 1.2× Ampure XP bead clean up, eluting in 20 μL. Quality control was performed on a Bio-analyzer HS. Target region enrichment was performed using the SeqCap Epi Choice S Enrichment Kit according to manufacturer’s guidelines from chapter 5 to 7. We combined uniquely indexed libraries by pooling up to 1000 ng (max. 8 **×** 125 ng). The bead-bound captured samples were eluted in 40 μL of water. The captured, bisulfite converted libraries were amplified in two 50 μL reactions. Quality control was performed on a Bio-analyzer HS. Paired-end sequencing was performed on a HiSeq4000 from Illumina. Target MethylSeq data analysis: TrimGalore was used to trim off 10 bp from the 3′- and 5′-end of all reads, to remove errors introduced by the adaptase technology of the Accel-NGS Methyl-Seq during library preparation. The trimmed FASTQ files were directionally aligned to a bisulfite converted reference genome (multi-seed length of 20 bp, maximally one mismatch allowed) and deduplicated using Bismark. Coverage files were extracted from the aligned, sorted, and deduplicated BAM files using Bismark’s methylation extractor. Further analysis was performed in R. Unless stated explicitly, methylation levels were calculated per gene promoter as the mean methylation level of all CpGs in the region 2000 bp upstream and 500 bp downstream of the gene’s transcription start site. ctDNA fraction estimation: to estimate which fraction of the total cfDNA originates from the tumor, we used the set of 47,569 CpGs that are lowly methylated in normal blood (defined above). Measured methylation levels at these sites were correlated with their corresponding methylation levels in pure tumor that were extracted from 370 HCC patients in the TCGA database. We assume that *M*_meas_ = *M*_tumor_ * f_ctDNA_ + *M*_cfDNA_ * f_cfDNA_, where f_ctDNA_ is the fraction of ctDNA, f_cfDNA_ is the fraction of other sources cfDNA, and f_ctDNA_ + f_cfDNA_ = 1. Thus, when *M*_cfDNA_~0, we can approach f_ctDNA_ by *M*_meas_/*M*_tumor_. This we calculated using linear modeling in R. For the final assessment of EMT in patient-derived liquid biopsy samples, 13 EMT markers were excluded based on their high methylation levels in the blood obtained from control samples. These markers are *WNT5A*, *VCAM1*, *SPARC*, *MMP9*, *GRHL2*, *CTNNB1*, *CTNNA1*, *COL8A2*, *COL5A2*, *COL4A2*, *COL3A1*, *COL1A2*, and *AXL.* All methylation measurements in the region 2000 bp upstream and 500 bp downstream of the transcription start sites of the remaining genes in the EMT gene signature (Additional file 1: Table S1) were used. Correlation between tissue and liquid biopsy methylation levels was estimated by pairwise comparison of obtained methylation levels of all target CpGs between the tissue and liquid biopsy samples. The Spearman rank order correlation coefficient was used, corrected for ctDNA fraction.

### Data analyses

All data processing was performed in R version 3.4.2. Bedtools was used to annotate CpGs to promoter regions which we defined as being 2000 bp upstream to 500 bp downstream of the transcription start site. The Bioconductor package minfi was used for methylation analysis.

### Statistical analyses

The R packages data.table, dplyr, Rmisc, and matrixStats were used for statistical analyses. Before testing if differences between two groups of data were significant, normality of underlying data was checked using the Shapiro test. Since most datasets failed normality check, all data was processed using unpaired, two-sided Mann-Whitney *U* test statistics unless when stated explicitly. For all *p* values below 0.05, the results were considered to be statistically significant. Correlations were calculated using Spearman rank order correlation coefficient. Bar plots show means with standard error of means. All boxplots show medians with interquartile ranges.

## Supplementary information

**Additional file 1:Table S1.** The EMT signature. **Table S2.** Number of overlapping CpGs between the indicated cell line models. **Table S3.** Expected amount of hyper- (lower half) and hypo- (upper half) methylated CpGs that are common between 2 EMT resistance models. **Table S4.** Significance pairwise overlap differentially methylated CpG sites. **Table S5.** IC_50_ values of all drugs included in this study for the parental cells. **Table S6.** Patients included in the clinical part of this study. **Table S7.** Correlation of DNA methylation data between all available tissue biopsies from various patients and the liquid biopsy data from patient 6. **Table S8.** Correlation of DNA methylation data between all available liquid biopsies from the various patients included in the study and the methylation data obtained from the tissue biopsy of patient 6.

**Additional file 2.** ‘Raw patient DNA methylation data of EMT marker genes promoter regions’, is provided as a comma separated. It is the result of our novel liquid biopsy approach (see Methods section) and contains from all subjects included in this study the raw DNA methylation data of EMT marker genes promoter regions.

**Additional file 3: Figure S1.** Characterization of the cell lines. (A) IC_50_ values of 10 pairs of parental and resistant cell lines determined by establishing dose-response curves using the SRB assay. IC_50_ values of parental cells are depicted on the y-axis in black, of resistant cells in red (Primary data available in Table 1). Names of resistant cell lines indicate the cell line pairs. (B) Boxplots of log_2_ transformed read counts of epithelial (16, gray) and mesenchymal (34, red) marker genes for all cell lines determined by RNA sequencing. Significance of the difference between epithelial and mesenchymal marker gene expression was calculated using a two-sided Mann-Whitney U test. (C) As in (B) for cells transfected with mock miRNA, determined using RT-qPCR and calculated using the delta-delta Ct method. Significance of the difference between epithelial and mesenchymal marker gene expression was calculated using a one-sided Mann-Whitney U test. (D) Growth of parental (black) and resistant (red) cells under drug pressure at the corresponding IC_50_ of the parental cells after transfection with mock miRNA, relative to mock miRNA transfected cells unexposed to the drugs. Shown are data from individual experiments (points) and the means (bars). Significance of the difference between parental and resistant cell growth was calculated using a one-sided Mann-Whitney U test. For all cell line pairs, *n* = 6, except for PancVB, PancB1Q and PancB1V, *n* = 3, each replication consisting of 9 technical replicates. ns: non-significant, *: *p*-value< 0.05, **: *p-*value< 0.01, ***: *p-*value< 0.001, ****: *p-*value< 0.0001

**Additional file 4:Figure S2.** Morphology of the cell line models. Pictures of all parental and resistant cell lines included in this study. Red boxes contour pictures from cell lines originating from the same parental cell line. All images are representative examples.

**Additional file 5:Figure S3.** Cell surface expression of EPCAM protein in all cell lines. Shown are signal intensities of APC-conjugated anti-EPCAM antibody (x-axis, log_10_ scale) and Forward Scatter Area (FSC-A; y-axis, linear scale), assessed by flow cytometry. Red boxes contour data panels from cell lines originating from the same parental cell line.

**Additional file 6:Figure S4.** Copy number aberration profiles of all cell lines used in this study. Depicted are logR values (y-axis) per genomic segment (x-axis) characterized by a uniform copy number profile. Red boxes contour profiles from cell lines originating from the same parental cell line.

**Additional file 7: Figure S5.** DNA methylation profiles correlate with EMT expression profiles (A) Fraction of hyper- and hypomethylated CpGs upon resistance acquirement. Red bar plots represent the fraction of interrogated CpGs (all CpGs included on the 450 K array) that are hypermethylated (with ß_parental_ < 0.3 and ß_resistant_ > 0.7) and blue bar plots the fraction of interrogated CpG that are hypomethylated (ß_parental_ > 0.7 and ß_resistant_ < 0.3) in resistant cells relative to parental cells. (B) Methylation changes upon resistance (Δß = ß_resistant_ - ß_parental_) of CpGs in epithelial (117) and mesenchymal (275) marker gene promoter regions represented by gray and red boxplots respectively. Significance of the difference between epithelial and mesenchymal promoter methylation changes was calculated using a one-sided Mann-Whitney U test. (C) Correlation of EMT marker gene expression with promoter methylation. On the x-axis, the methylation difference of each CpG in EMT marker gene promoters between resistant and parental cells is depicted. On the y-axis, the log_2_ (fold-change) expression difference between resistant and parental cells of the corresponding EMT marker gene is depicted. In EMT models, mesenchymal gene promoter CpGs (red dots) are expected in the upper left quadrant and epithelial gene promoter CpGs (gray dots) in the lower right quadrant. (D) Fraction of epithelial (gray bar plots) and mesenchymal (red bar plots) genes that lie respectively in the lower right and upper left quadrant of Fig. 2b. The dotted line indicates the expected fraction (0.25). (E) Fraction of epithelial (gray bar plots) and mesenchymal (red bar plots) CpGs that lie respectively in the lower right and upper left quadrant of panel C. The dotted line indicates the expected fraction (0.25). ns: non-significant, *: *p*-value< 0.05, **: *p-*value< 0.01, ***: *p-*value< 0.001, ****: *p-*value< 0.0001.

**Additional file 8: Figure S6.** Control experiments concerning pharmacological demethylation of the cell line models. (A) Global cytosine methylation levels in parental (black) and resistant (red) cells determined by LC/MS before (no contour) and after (yellow contour) an 8-day exposure to a non-cytotoxic dose (0.5 μM) of the pharmacologically demethylating agent 5-aza-2′-deoxycytidine. Methylated cytosine levels are expressed relatively to all cytosines. Names of resistant lines are used to indicate the cell pairs. (B) EMT marker gene expression difference between resistant and parental cells after an 8-day vehicle (DMSO) treatment. Boxplots depict log_2_(fold-change) expression differences between resistant and parental cells of 16 epithelial (gray) and 34 mesenchymal (red) marker genes determined by RT-qPCR and calculated using the delta-delta Ct method. Significance of the difference between epithelial and mesenchymal gene expression changes was calculated using a one-sided Mann-Whitney U test. (C) Cell growth of parental (black) and resistant (red) cells after an 8-day vehicle (DMSO) treatment under drug pressure at the estimated IC_50_ of the parental cells (Primary data in Table 1) is depicted on the y-axis, expressed relative to growth of DMSO-pretreated cells not exposed to the drugs. Shown are data from individual experiments (points) and the means (bars). Experiments were performed in triplicate (except for IGROV-1/CDDP, *n* = 2), each replication consisting of 9 technical replicates. Significance of the difference between parental and resistant cell growth was calculated using a one-sided Mann-Whitney U test. ns: non-significant, *: *p*-value< 0.05, **: *p-*value< 0.01, ***: *p-*value< 0.001, ****: *p-*value< 0.0001.

**Additional file 9: Figure S7.** Effect of the inhibition of the DNA methyl transferases on EMT-mediated resistance in 3 model cell line pairs. (A) The change in mRNA levels of *DNMT1, DNMT3A* and *DNMT3B* after DNMT knock down, assessed through qPCR. *DNMT* gene expression by knock down cells is depicted as gray bar plots (y-axis), relative to expression of cells transfected with non-targeting control siRNAs (white bar plots). Standard errors of means are indicated. Results stem from *n* = 3 biological replicates. Significance of the difference between control and knock down expression was calculated using a one-sided Mann-Whitney U test. (B) As in A, for DNMT1 and DNMT3A protein levels, assessed through western blotting. (C) Representative western blot images of DNMT protein expression in knock down cells as well as in cells transfected with non-targeting control siRNAs. Beta-actin was used as a loading control. (D) EMT marker gene expression difference between resistant and parental cells after transfection with non-targeting control siRNAs or siRNAs targeting *DNMT1, DNMT3A* and *DNMT3B*. Boxplots depict log2 (fold change) expression differences between resistant and parental cells of 16 epithelial (gray) and 34 mesenchymal (red) marker genes, as determined by RNA-seq. Significance of gene expression differences was calculated using one-sided Mann-Whitney U tests. (E) Cell growth of parental (black) and resistant (red) cells after transfection with control non-targeting siRNA pools or siRNA pools targeting *DNMT1, DNMT3A* and *DNMT3B* under drug pressure at the estimated IC50 of the parental cells (Primary data in Table 1) is depicted on the y-axis, expressed relative to growth of transfected cells not exposed to the drugs. Shown are data from individual experiments (points) and the means (bars). Experiments were performed in triplicate (except for HepG2 and HepG2S3, *n* = 4), each replication consisting of at least 9 technical replicates. Significance of the difference between parental and resistant cell growth was calculated using a one-sided Mann-Whitney U test. ns: non-significant, *: *p-*value< 0.05, **: *p-*value< 0.01, ***: *p-*value< 0.001, ****: *p-*value< 0.0001.

**Additional file 10: Figure S8.** Expression of the DNA methyltransferases on RNA and protein level. (A) Gene expression levels of *DNMT1, DNMT3A* and *DNMT3B* (shades of blue) genes in both parental and resistant cell lines are depicted as bar plots (y-axis, log_2_ normalized counts (counts per million, CPM) from RNA sequencing data (*n* = 1)). As a reference, gene expression levels of housekeeper gene *ACTB* are depicted as red bar plots. (B) Western blots showing protein expression of DNMT1 and DNMT3A in both parental and resistant cell lines. Beta-actin was used as a loading control. (C) Protein expression of DNMT1 and DNMT3A in both parental and resistant cell lines quantified from western blot band intensities depicted as bar plots (y-axis, relative to average protein expression of parental cells). Standard errors of means are indicated from at least n = 3 biological replicates. Statistical testing revealed no significant difference in expression between parental and resistant cell lines for either DNMT1 or DNMT3A expression.

**Additional file 11:Figure S9.** (A) Gene expression levels of all TET (shades of blue) genes in both parental and resistant cell lines are depicted as bar plots (y-axis, log_2_ normalized counts (counts per million, CPM) from RNA sequencing data (n = 1)). As a reference, gene expression levels of housekeeper gene ACTB are depicted in red bar plots. (B) Hydroxymethylation of CpGs binned according to methylation levels. Distributed over the x-as, all CpGs contained on Illumina’s 450 K methylation array are divided into 10 bins according to their methylation levels. In each bin, hydroxymethylation levels of hyper- or hypomethylated CpGs are depicted in red and blue boxplots respectively and compared to CpGs with stable methylation levels which are depicted in gray boxplots. Bins 4 to 7 only contain CpGs with stable methylation between parental and resistant cells, hence no comparison is possible.

**Additional file 12:Figure S10.** Methylation in control blood samples. (A) Bar plots depict methylation levels (%) of all CpGs included on the custom probes (in salmon) and of target CpGs (in green) for all 16 control samples. Shown are means ± SEM. (B) Probe CpG methylation levels (%) as measured by our novel technique in the first control blood sample (y-axis) plotted against published methylation levels of corresponding CpGs assessed in normal blood from a healthy male subject using WGBS (GEO accession number GSM1091963 [34], x-axis, %). (C) Linear model coefficients (direction coefficient and Spearman R^2^) of the correlation depicted in panel B for all control samples. (D) Pairwise correlation between tissue biopsies methylation results and the methylation results of the liquid biopsies. Thickness of the connection indicates the corresponding Spearman correlation R of the connected biopsies (Primary data available in (Additional file 1: Tables S7 and S8)). (E) For each EMT marker gene (y-axis), promoter methylation is calculated as the average methylation of all its CpGs (data pooled from all control samples) and depicted as bar plots (x-axis). Shown are means ± SEM. The dotted line indicates EMT marker genes that are considered too highly and/or variably methylated in normal blood and are therefore excluded for further analyses in this study. (F) Boxplots depict CpG methylation levels (%) of all EMT marker gene promoter regions, from all 16 control samples.

## Data Availability

The RNA sequencing, DNA methylation, and hydroxymethylation datasets supporting the conclusions of this article are available in the Gene Expression Omnibus (GEO) repository under the accession number GSE128686, https://www.ncbi.nlm.nih.gov/geo/query/acc.cgi?acc=GSE128686 and GSE142586, https://www.ncbi.nlm.nih.gov/geo/query/acc.cgi?acc=GSE142586. The low coverage, whole genome sequencing data supporting copy number analysis of all cell lines has been deposited at https://www.ncbi.nlm.nih.gov/bioproject/595921 under the SRA accession number PRJNA595921. All tabular data supporting the conclusions of this article that are not presented in the main manuscript are included in Additional file 1. The targeted bisulfite sequencing data of EMT promoter regions of all patients supporting conclusions based on Fig. 5 are included in Additional file 2. The authors declare that other data supporting the findings of this study not available within the article or it additional files, can be delivered upon reasonable request.

## Abbreviations

(q)PCR: (quantitative) Polymerase chain reaction
450K: Infinium humanmethylation450 beadchip
5hmC: 5-Hydroxymethylcytosine
5mC: 5-Methylcytosine
aza: 5-Aza-2′-deoxycytidine
C: Cytosine
cfDNA: Cell-free DNA
CpG: Cytosine guanine dinucleotide
C_t_: Cycle threshold
ctDNA: Circulating tumor DNA
DFO: Deferoxamine
DMSO: Dimethylsulfoxide
DNMT: DNA methyl transferase enzyme
EMT: Epithelial-to-mesenchymal transition
EPIC: Infinium HumanMethylationEPIC BeadChips
FBS: Fetal bovine serum
f_cfDNA_: Fraction cfDNA
f_ctDNA_: Fraction ctDNA
H_2_O: Water
HCC: Hepatocellular carcinoma
IC_50_: Inhibitory concentration 50
LC/MS: Liquid chromatography/mass spectrometry
Log_2_FC: Log_2_ fold change
M_cfDNA_: Methylation level of cfDNA
miR-200: MicroRNA 200
miRNA: MicroRNA
*M*_meas_: Methylation level measured in plasma
*M*_tumor_: Methylation level of ctDNA
PS: Penicillin-streptomycin
SRB assay: Sulforhodamine B Colorimetric assay
SWAN: Subset-quantile within array normalization
TAB-ChIP: TET-assisted bisulfite chromatin immunoprecipitation
TAB-Seq: TET-assisted bisulfite sequencing
TCGA: The Cancer Genome Atlas
TET: Ten-eleven translocation enzyme
TGF-B: Transforming growth factor-beta
