# Supplementary material for: DNA methylation-driven EMT is a common mechanism of resistance to various therapeutic agents in cancer
Source: Clin Epigenetics. 2020 Feb 14;12:27. doi: 10.1186/s13148-020-0821-z (PMC7023776; doi:10.1186/s13148-020-0821-z)
Supplement: Supplementary file 1 — Additional file 1:Table S1. The EMT signature. Table S2. Number of overlapping CpGs between the indicated cell line models. Table S3. Expected amount of hyper- (lower half) and hypo- (upper half) methylated CpGs that are common between 2 EMT resistance models. Table S4. Significance pairwise overlap differentially methylated CpG sites. Table S5. IC50 values of all drugs included in this study for the parental cells. Table S6. Patients included in the clinical part of this study. Table S7. Correlation of DNA methylation data between all available tissue biopsies from various patients and the liquid biopsy data from patient 6. Table S8. Correlation of DNA methylation data between all available liquid biopsies from the various patients included in the study and the methylation data obtained from the tissue biopsy of patient 6. [file 13148_2020_821_MOESM1_ESM.docx]

# Supplementary Tables

DNA methylation-driven EMT is a common mechanism of resistance to various therapeutic agents in cancer

**Authors:** Eva Galle^1,2,3^, Bernard Thienpont^3^, Sarah Cappuyns^1,2,4^, Tom Venken^1,2^, Pieter Busschaert^1,2^, Matthias Van Haele^5^, Eric Van Cutsem^4^, Tania Roskams^5^, Jos van Pelt^6^, Chris Verslype^4^, Jeroen Dekervel^4,*^, Diether Lambrechts^1,2,*^

**Affiliations:**

^1^ Centre for Cancer Biology, VIB, 3000 Leuven, Belgium
^2^ Laboratory of Translational Genetics, Department of Human Genetics, KU Leuven, 3000 Leuven, Belgium

^3^ Laboratory for Functional Epigenetics, Department of Human Genetics, KU Leuven, 3000 Leuven, Belgium

^4^ Clinical Digestive Oncology, Department of Oncology, KU Leuven and University Hospitals Leuven, 3000 Leuven, Belgium

^5^ Department of Imaging and Pathology, Translational Cell and Tissue Research, KU Leuven and University Hospitals Leuven, 3000 Leuven, Belgium

^6^ Laboratory of Clinical Digestive Oncology, Department of Oncology, KU Leuven and Leuven Cancer Institute (LKI), 3000 Leuven, Belgium

^*^ Corresponding author

**Table S1**. The EMT signature.

| **GeneID** | **Status** | **Reference** | **GeneID** | **Status** | **Reference** |
| --- | --- | --- | --- | --- | --- |
| *CDH1* | Epi | (1-5) | *LAD1* | Epi | (5) |
| *CDH2* | Mes | (1-3, 5) | *LOXL2* | Mes | (6, 7) |
| *COL11A1* | Mes | (8, 9) | *MMP2* | Mes | (10-12) |
| *COL12A1* | Mes | (7, 13) | *MMP9* | Mes | (10-12) |
| *COL1A1* | Mes | (5, 12) | *MSN* | Mes | (4) |
| *COL1A2* | Mes | (4, 9) | *NOTCH2* | Mes | (14, 15) |
| *COL3A1* | Mes | (4, 5, 9, 16) | *OCLN* | Epi | (5, 16, 17) |
| *COL4A1* | Mes | (12, 13) | *PLAUR* | Mes | (11, 12) |
| *COL4A2* | Mes | (12, 13) | *PRRX1* | Mes | (9, 18) |
| *COL5A1* | Mes | (5, 9, 16) | *PRSS2* | Mes | (7) |
| *COL5A2* | Mes | (4, 9, 13) | *SNAI1* | Mes | (17, 19, 20) |
| *COL5A3* | Mes | (7) | *SNAI2* | Mes | (10, 20, 21) |
| *COL6A2* | Mes | (9, 16) | *SPARC* | Mes | (10, 20, 21) |
| *COL7A1* | Mes | (7, 12) | *SPINT1* | Epi | (5, 22) |
| *COL8A2* | Mes | (7, 23) | *TGF-B* | Mes | (24, 25) |
| *CTNNA1* | Epi | (26) | *TWIST1* | Mes | (10, 20) |
| *CTNNB1* | Epi | (4) | *VCAM1* | Mes | (27, 28) |
| *DSP* | Epi | (20, 29) | *VCAN* | Mes | (5, 9, 12, 30) |
| *EPCAM* | Epi | (5, 28) | *VEGFA* | Mes | (12, 31, 32) |
| *FN1* | Mes | (1-3, 5) | *VEGFC* | Mes | (7, 33) |
| *GRHL2* | Epi | (8, 28, 34, 35) | *VIM* | Mes | (1-3) |
| *GSK3B* | Epi | (36, 37) | *WNT5A* | Mes | (5, 38, 39) |
| *KLF5* | Epi | (40) | *YAP1* | Mes | (41) |
| *KRT18* | Epi | (4, 10, 23) | *ZEB1* | Mes | (1, 10, 20) |
| *KRT19* | Epi | (4, 10, 20, 28) | *ZEB2* | Mes | (1, 10, 20) |

Genes that were used in this study to assess EMT on a transcriptional level together with their OpenArray ID, epithelial (‘Epi’) or mesenchymal (‘Mes’) nature and reference.

**Table S2.** Number of overlapping CpGs between the indicated cell line models.

|  | **HepG2S1** | **HepG2S3** | **PancB1Q** | **PancB1V** | **HCC4006ER** | **HCC4006GR** | **HCC827GR** |
| --- | --- | --- | --- | --- | --- | --- | --- |
| **HepG2S1** |  | 6512 | 56 | 70 | 65 | 20 | 23 |
| **HepG2S3** | 38474 |  | 69 | 90 | 93 | 30 | 30 |
| **PancB1Q** | 1013 | 1009 |  | 1021 | 26 | 1 | 4 |
| **PancB1V** | 611 | 610 | 3000 |  | 29 | 4 | 6 |
| **HCC4006ER** | 1352 | 1353 | 358 | 200 |  | 1 | 14 |
| **HCC4006GR** | 14 | 16 | 5 | 3 | 4 |  | 11 |
| **HCC827GR** | 26 | 27 | 10 | 9 | 29 | 4 |  |

Total number of hyper- (lower half) and hypo- (upper half) methylated CpGs shared pairwise between the EMT cell line models.

**Table S3.** Expected amount of hyper- (lower half) and hypo- (upper half) methylated CpGs that are common between 2 EMT resistance models.

|  | **HepG2S1** | **HepG2S3** | **PancB1Q** | **PancB1V** | **HCC4006ER** | **HCC4006GR** | **HCC827GR** |
| --- | --- | --- | --- | --- | --- | --- | --- |
| **HepG2S1** |  | 142 | 17 | 21 | 22 | 2 | 9 |
| **HepG2S3** | 4044 |  | 22 | 28 | 29 | 3 | 12 |
| **PancB1Q** | 448 | 455 |  | 3 | 3 | 0 | 1 |
| **PancB1V** | 270 | 273 | 30 |  | 4 | 0 | 1 |
| **HCC4006ER** | 669 | 678 | 75 | 29 |  | 0 | 2 |
| **HCC4006GR** | 8 | 8 | 1 | 1 | 1 |  | 0 |
| **HCC827GR** | 13 | 13 | 1 | 1 | 2 | 0 |  |

Expected amounts were calculated by multiplying the product of the frequencies of hyper-/hypomethylation events (=amount of hyper-/hypomethylated CpGs divided by the number of interrogated sites on the assay) seen in each of the pair by the amount of measurements (=number of interrogated sites on the assay).

**Table S4.** Significance pairwise overlap differentially methylated CpG sites.

|  | **HepG2S1** | **HepG2S3** | **PancB1Q** | **PancB1V** | **HC4006ER** | **HCC4006GR** | **HCC827GR** |
| --- | --- | --- | --- | --- | --- | --- | --- |
| **HepG2S1** |  | 0 | 3.50E-13 | 6.43E-15 | 8.14E-12 | 9.18E-13 | 3.70E-05 |
| **HepG2S3** | 0 | 0 | 1.60E-14 | 5.40E-19 | 5.32E-18 | 9.68E-20 | 4.17E-06 |
| **PancB1Q** | 2.45E-95 | 1.07E-89 |  | 0 | 2.02E-12 | 0.303666 | 0.0481873 |
| **PancB1V** | 4.07E-58 | 2.66E-55 | 0 | 0 | 3.91E-12 | 0.0011912 | 0.0074972 |
| **HCC4006ER** | 4.68E-98 | 8.88E-94 | 7.15E-107 | 3.92E-57 |  | 0.3734777 | 5.43E-09 |
| **HCC4006GR** | 0.0237301 | 0.050706 | 0.001877 | 0.015454 | 0.0405374 | 0 | 0.0009570 |
| **HCC827GR** | 0.0007439 | 0.000421 | 2.78E-06 | 3.36E-07 | 5.39E-23 | 1 |  |

P-values resulting from binomial test investigating whether the amount of common hyper- (lower half) and hypo- (upper half) methylated CpGs (in Supplementary Table 2) is significantly different from the amount of expected shared CpGs (in Supplementary Table 3).

**Table S5**. IC_50_ values of all drugs included in this study for the parental cells

| Parental Cell Line | Sorafenib | 5-Fluorouracil | Erlotinib | Gefitinib | Olaparib | Cisplatin |
| --- | --- | --- | --- | --- | --- | --- |
| HepG2 | 0.28 | 0.08 | 0.12 | 256.00 | 22.63 | 16.00 |
| PancVB | 11.62 | 14.63 | 12.13 | 21.86 | 1541.37 | 161.90 |
| HCC4006P1 | 10.46 | 18.64 | 0.03 | 0.12 | 256.00 | 98.16 |
| HCC4006P2 | 21.68 | 66.23 | 0.02 | 0.10 | 80.00 | 362.04 |
| HCC827GR | 30.61 | 25.42 | 0.01 | 0.03 | 5.07 | 13.00 |
| UWB.1.289 | 0.31 | 32.00 | 0.14 | 40.80 | 58.02 | 12.91 |
| IGROV-1 | 8.18 | 4.43 | 4.64 | 8.23 | 15.78 | 64.04 |

Concentrations (µM) at which parental cell growth is estimated to be inhibited by 50% for all cell lines against all drugs used in this study. Concentrations were used for assessing cross resistance and cross resistance loss after demethylation in Figure 4.

**Table S6**. Patients included in the clinical part of this study.

| **Patient** | **Resistance** | **Days to second sample** | **Response at second sample** | **Days to last sample** | **Response at last sample** |
| --- | --- | --- | --- | --- | --- |
| 1 | Acquired | 70 | PR | 210 | PD |
| 2 | Acquired | 48 | Mixed | 85 | PD |
| 3 | Acquired | 56 | Mixed | 315 | PD |
| 4 | Acquired | 56 | PR | 168 | PD |
| 5 | Acquired | 119 | SD | 371 | PD |
| 6 | Acquired | 51 | Mixed | 73 | PD |
| 7 | / | / | / | 448 | Response |
| 8 | / | / | / | 546 | Response |
| 9 | / | / | / | 42 | Response |
| 10 | Intrinsic | / | / | 45 | PD |
| 11 | Intrinsic | / | / | 64 | PD |
| 12 | Intrinsic | / | / | 104 | PD |

The columns contain patient identification, type of resistance, days until the intermediate sample was taken, response as assessed by the clinician at the time of the intermediate biopsy, days until the last sample was taken and response as assessed by the clinician at the time of the last biopsy. PD=progressive disease, PR=partial response, SD=stable disease.

**Table S7**. Correlation of DNA methylation data between all available tissue biopsies from various patients and the liquid biopsy data from patient 6.

|  | **Spearman R to cfDNA Patient 6_A** | | **Spearman R to cfDNA Patient 6_B** | **Spearman R to cfDNA Patient 6_C** |
| --- | --- | --- | --- | --- |
| Patient 6 | | 0.21 | 0.23 | 0.26 |
| Patient 13 | | 0.19 | 0.19 | 0.20 |
| Patient 14 | | 0.18 | 0.17 | 0.19 |
| Patient15 | | 0.21 | 0.21 | 0.22 |
| Patient 16 | | 0.19 | 0.19 | 0.21 |

Spearman R values for each pairwise correlation between tissue biopsies methylation results and the methylation results of the liquid biopsies of patient 6, for whom matched liquid and tissue biopsy are available.

**Table S8**. Correlation of DNA methylation data between all available liquid biopsies from the various patients included in the study and the methylation data obtained from the tissue biopsy of patient 6.

| **Patients** | **Spearman R to fixed tissue Patient 6** |
| --- | --- |
| Patient 1_A | 0.07 |
| Patient 1_B | 0.12 |
| Patient 1_E | 0.02 |
| Patient 2_A | 0.17 |
| Patient 2_B | 0.23 |
| Patient 2_C | 0.11 |
| Patient 3_A | 0.12 |
| Patient 3_B | 0.03 |
| Patient 3_C | 0 |
| Patient 4_B | 0.06 |
| Patient 4_C | 0.12 |
| Patient 4_E | 0.16 |
| Patient 5_A | 0.05 |
| Patient 5_B | 0.18 |
| Patient 5_F | 0.14 |
| Patient 6_A | 0.14 |
| Patient 6_B | 0.23 |
| Patient 6_C | 0.21 |
| Patient 7_A | 0.12 |
| Patient 7_G | 0 |
| Patient 8_A | 0.15 |
| Patient 8_I | 0.19 |
| Patient 9_A | 0.14 |
| Patient 9_C | 0.15 |
| Patient 10_A | 0.04 |
| Patient 10_B | 0.03 |
| Patient 11_A | 0.05 |
| Patient 11_B | 0.14 |
| Patient 12_A | 0.11 |
| Patient 12_B | 0.07 |

Spearman R values for each pairwise correlation between liquid biopsies methylation results and the methylation results of the tissue biopsy of patient 6. for whom matched liquid and tissue biopsy are available.

# References

1. Gemmill RM, Roche J, Potiron VA, Nasarre P, Mitas M, Coldren CD, et al. ZEB1-responsive genes in non-small cell lung cancer. Cancer letters. 2011;300(1):66-78.

2. Yauch RL, Januario T, Eberhard DA, Cavet G, Zhu W, Fu L, et al. Epithelial versus Mesenchymal Phenotype Determines In vitro Sensitivity and Predicts Clinical Activity of Erlotinib in Lung Cancer Patients. Clinical Cancer Research. 2005;11(24):8686-98.

3. Thomson S, Buck E, Petti F, Griffin G, Brown E, Ramnarine N, et al. Epithelial to Mesenchymal Transition Is a Determinant of Sensitivity of Non–Small-Cell Lung Carcinoma Cell Lines and Xenografts to Epidermal Growth Factor Receptor Inhibition. Cancer Research. 2005;65(20):9455-62.

4. Chang H, Liu Y, Xue M, Liu H, Du S, Zhang L, et al. Synergistic action of master transcription factors controls epithelial-to-mesenchymal transition. Nucleic Acids Research. 2016;44(6):2514-27.

5. Gröger CJ, Grubinger M, Waldhör T, Vierlinger K, Mikulits W. Meta-analysis of gene expression signatures defining the epithelial to mesenchymal transition during cancer progression. PloS ONE. 2012;7(12):e51136.

6. Cuevas EP, Eraso P, Mazón MJ, Santos V, Moreno-Bueno G, Cano A, et al. LOXL2 drives epithelial-mesenchymal transition via activation of IRE1-XBP1 signalling pathway. Scientific Reports. 2017;7:44988.

7. Liberzon A, Birger C, Thorvaldsdóttir H, Ghandi M, Mesirov Jill P, Tamayo P. The Molecular Signatures Database Hallmark Gene Set Collection. Cell Systems. 2015;1(6):417-25.

8. García-Pravia C, Galván JA, Gutiérrez-Corral N, Solar-García L, García-Pérez E, García-Ocaña M, et al. Overexpression of COL11A1 by Cancer-Associated Fibroblasts: Clinical Relevance of a Stromal Marker in Pancreatic Cancer. PLoS ONE. 2013;8(10):e78327.

9. Cheng W-Y, Kandel JJ, Yamashiro DJ, Canoll P, Anastassiou D. A Multi-Cancer Mesenchymal Transition Gene Expression Signature Is Associated with Prolonged Time to Recurrence in Glioblastoma. PLoS ONE. 2012;7(4):e34705.

10. Sarrió D, Rodriguez-Pinilla SM, Hardisson D, Cano A, Moreno-Bueno G, Palacios J. Epithelial-Mesenchymal Transition in Breast Cancer Relates to the Basal-like Phenotype. Cancer Research. 2008;68(4):989-97.

11. Santibanez JF, Obradović H, Kukolj T, Krstić J. Transforming Growth Factor-beta, Matrix Metalloproteinases and Urokinase-Type Plasminogen Activator Interaction in the Cancer Epithelial to Mesenchymal Transition: TGF-β, MMPs and uPA interplay in cancer EMT. Dev Dyn. 2017;247:382-95.

12. Reka AK, Chen G, Jones RC, Amunugama R, Kim S, Karnovsky A, et al. Epithelial-mesenchymal transition-associated secretory phenotype predicts survival in lung cancer patients. Carcinogenesis. 2014;35(6):1292-300.

13. Roson-Burgo B, Sanchez-Guijo F, Del Cañizo C, De Las Rivas J. Insights into the human mesenchymal stromal/stem cell identity through integrative transcriptomic profiling. BMC genomics. 2016;17(1):944-.

14. Hayashi T, Gust KM, Wyatt AW, Goriki A, Jäger W, Awrey S, et al. Not all NOTCH Is Created Equal: The Oncogenic Role of NOTCH2 in Bladder Cancer and Its Implications for Targeted Therapy. Clinical Cancer Research. 2016;22(12):2981-92.

15. Timmerman LA, Grego-Bessa J, Raya A, Bertrán E, Pérez-Pomares JM, Díez J, et al. Notch promotes epithelial-mesenchymal transition during cardiac development and oncogenic transformation. Genes & development. 2004;18(1):99-115.

16. Fischer KR, Durrans A, Lee S, Sheng J, Li F, Wong ST, et al. Epithelial-to-mesenchymal transition is not required for lung metastasis but contributes to chemoresistance. Nature. 2015;527(1):472-6.

17. Ikenouchi J, Matsuda M, Furuse M, Tsukita S. Regulation of tight junctions during the epithelium-mesenchyme transition: direct repression of the gene expression of claudins/occludin by Snail. Journal of Cell Science. 2003;116(10):1959-67.

18. Guo J, Fu Z, Wei J, Lu W, Feng J, Zhang S. PRRX1 promotes epithelial–mesenchymal transition through the Wnt/β-catenin pathway in gastric cancer. Medical Oncology. 2014;32(1):393.

19. Cano A, Pérez-Moreno MA, Rodrigo I, Locascio A, Blanco MJ, del Barrio MG, et al. The transcription factor Snail controls epithelial–mesenchymal transitions by repressing E-cadherin expression. Nature Cell Biology. 2000;2:76-83.

20. Nozato M, Kaneko S, Nakagawara A, Komuro H. Epithelial-mesenchymal transition-related gene expression as a new prognostic marker for neuroblastoma. International journal of oncology. 2012;42(1):134-40.

21. Fenouille N, Tichet M, Dufies M, Pottier A, Mogha A, Soo JK, et al. The Epithelial-Mesenchymal Transition (EMT) Regulatory Factor SLUG (SNAI2) Is a Downstream Target of SPARC and AKT in Promoting Melanoma Cell Invasion. PLoS ONE. 2012;7(7):e40378.

22. Cheng H, Fukushima T, Takahashi N, Tanaka H, Kataoka H. Hepatocyte Growth Factor Activator Inhibitor Type 1 Regulates Epithelial to Mesenchymal Transition through Membrane-Bound Serine Proteinases. Cancer Research. 2009;69(5):1828-35.

23. Aiello NM, Maddipati R, Norgard RJ, Balli D, Li J, Yuan S, et al. EMT Subtype Influences Epithelial Plasticity and Mode of Cell Migration. Developmental Cell. 2018;45(6):681-95.

24. Xu J, Lamouille S, Derynck R. TGF-β-induced epithelial to mesenchymal transition. Cell Research. 2009;19:156-72.

25. Zhang J, Tian X-J, Zhang H, Teng Y, Li R, Bai F, et al. TGF-β–induced epithelial-to-mesenchymal transition proceeds through stepwise activation of multiple feedback loops. Science Signaling. 2014;7(345):ra91.

26. Lee B, Villarreal-Ponce A, Fallahi M, Ovadia J, Sun P, Yu Q-C, et al. Transcriptional mechanisms link epithelial plasticity to adhesion and differentiation of epidermal progenitor cells. Developmental cell. 2014;29(1):47-58.

27. Wang P-C, Weng C-C, Hou Y-S, Jian S-F, Fang K-T, Hou M-F, et al. Activation of VCAM-1 and its associated molecule CD44 leads to increased malignant potential of breast cancer cells. International journal of molecular sciences. 2014;15(3):3560-79.

28. Tan TZ, Miow QH, Miki Y, Noda T, Mori S, Huang RY-J, et al. Epithelial-mesenchymal transition spectrum quantification and its efficacy in deciphering survival and drug responses of cancer patients. EMBO molecular medicine. 2014;6(10):1279-93.

29. Boelens MC, van den Berg A, Vogelzang I, Wesseling J, Postma DS, Timens W, et al. Differential expression and distribution of epithelial adhesion molecules in non-small cell lung cancer and normal bronchus. Journal of clinical pathology. 2007;60(6):608-14.

30. Arslan F, Bosserhoff AK, Nickl-Jockschat T, Doerfelt A, Bogdahn U, Hau P. The role of versican isoforms V0/V1 in glioma migration mediated by transforming growth factor-beta2. British journal of cancer. 2007;96(10):1560-8.

31. Weng C-H, Chen L-Y, Lin Y-C, Shih J-Y, Lin Y-C, Tseng R-Y, et al. Epithelial-mesenchymal transition (EMT) beyond EGFR mutations per se is a common mechanism for acquired resistance to EGFR TKI. Oncogene. 2018:455-68.

32. Gonzalez-Moreno O, Lecanda J, Green JE, Segura V, Catena R, Serrano D, et al. VEGF elicits epithelial-mesenchymal transition (EMT) in prostate intraepithelial neoplasia (PIN)-like cells via an autocrine loop. Experimental Cell Research. 2010;316(4):554-67.

33. Yeh Y-W, Cheng C-C, Yang S-T, Tseng C-F, Chang T-Y, Tsai S-Y, et al. Targeting the VEGF-C/VEGFR3 axis suppresses Slug-mediated cancer metastasis and stemness via inhibition of KRAS/YAP1 signaling. Oncotarget. 2016;8(3):5603-18.

34. Xiang J, Fu X, Ran W, Wang Z. Grhl2 reduces invasion and migration through inhibition of TGFβ-induced EMT in gastric cancer. Oncogenesis. 2017;6(1):e284.

35. Chung VY, Tan TZ, Tan M, Wong MK, Kuay KT, Yang Z, et al. GRHL2-miR-200-ZEB1 maintains the epithelial status of ovarian cancer through transcriptional regulation and histone modification. Scientific Reports. 2016;6:19943.

36. Kao SH, Wang WL, Chen CY, Chang YL, Wu YY, Wang YT, et al. GSK3β controls epithelial–mesenchymal transition and tumor metastasis by CHIP-mediated degradation of Slug. Oncogene. 2017;33(24):3172-82.

37. Liu Z-c, Wang H-s, Zhang G, Liu H, Chen X-h, Zhang F, et al. AKT/GSK-3β regulates stability and transcription of snail which is crucial for bFGF-induced epithelial–mesenchymal transition of prostate cancer cells. Biochimica et Biophysica Acta (BBA) - General Subjects. 2014;1840(10):3096-105.

38. Kanzawa M, Semba S, Hara S, Itoh T, Yokozaki H. WNT5A is a Key Regulator of the Epithelial-Mesenchymal Transition and Cancer Stem Cell Properties in Human Gastric Carcinoma Cells. Pathobiology. 2013;80(5):235-44.

39. Wang B, Tang Z, Gong H, Zhu L, Liu X. Wnt5a promotes epithelial-to-mesenchymal transition and metastasis in non-small-cell lung cancer. Bioscience reports. 2017;37(6):BSR20171092.

40. Zhang B, Zhang Z, Xia S, Xing C, Ci X, Li X, et al. KLF5 Activates MicroRNA 200 Transcription To Maintain Epithelial Characteristics and Prevent Induced Epithelial-Mesenchymal Transition in Epithelial Cells. Molecular and Cellular Biology. 2013;33(24):4919-35.

41. Shao Diane D, Xue W, Krall Elsa B, Bhutkar A, Piccioni F, Wang X, et al. KRAS and YAP1 Converge to Regulate EMT and Tumor Survival. Cell. 2014;158(1):171-84.
