# Supplementary figures and images for: DNA methylation-driven EMT is a common mechanism of resistance to various therapeutic agents in cancer
Source: Clin Epigenetics. 2020 Feb 14;12:27. doi: 10.1186/s13148-020-0821-z (PMC7023776; doi:10.1186/s13148-020-0821-z)

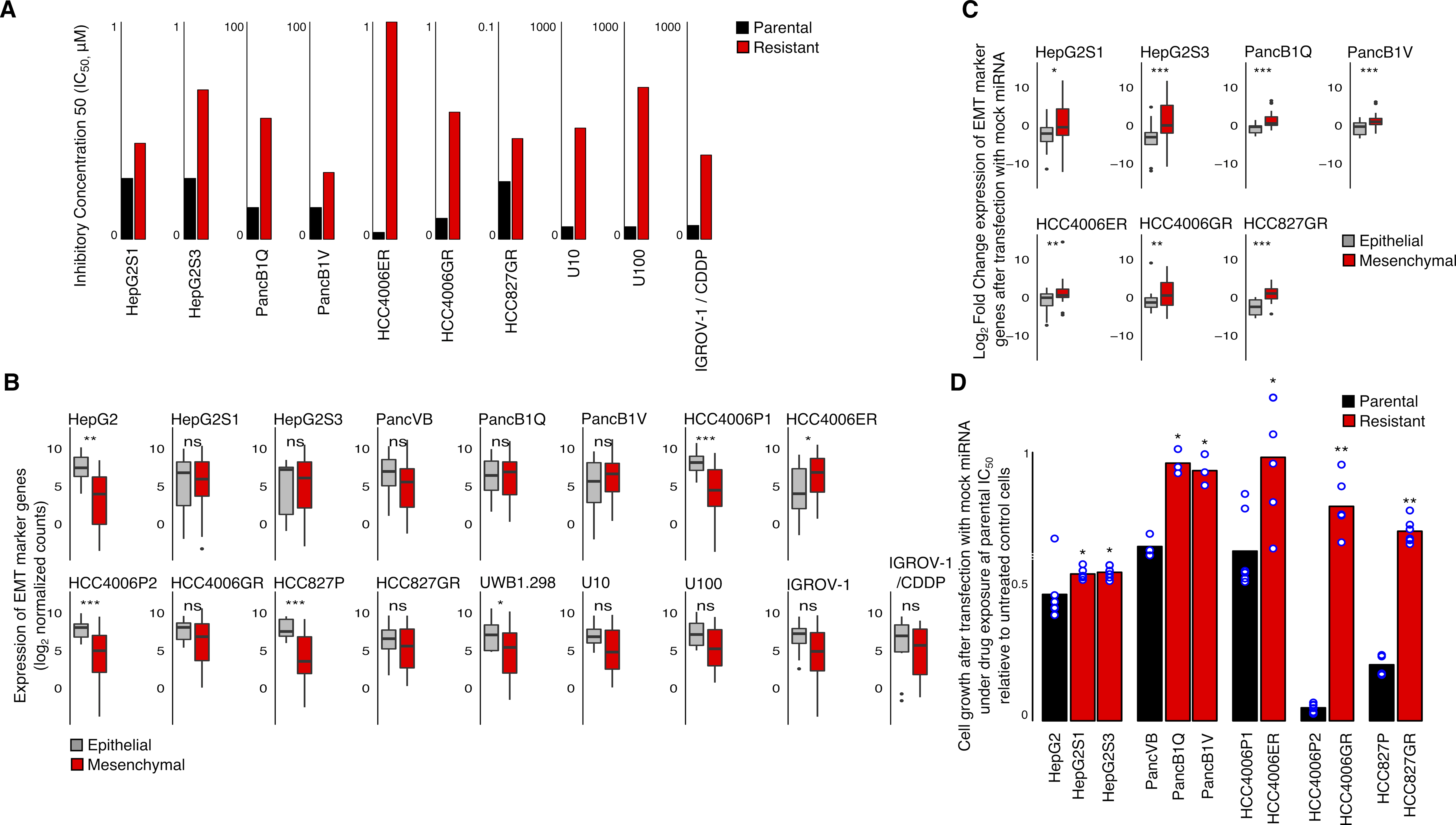

Supplement: Supplementary file 3 — Additional file 3: Figure S1. Characterization of the cell lines. (A) IC50 values of 10 pairs of parental and resistant cell lines determined by establishing dose-response curves using the SRB assay. IC50 values of parental cells are depicted on the y-axis in black, of resistant cells in red (Primary data available in Table 1). Names of resistant cell lines indicate the cell line pairs. (B) Boxplots of log2 transformed read counts of epithelial (16, gray) and mesenchymal (34, red) marker genes for all cell lines determined by RNA sequencing. Significance of the difference between epithelial and mesenchymal marker gene expression was calculated using a two-sided Mann-Whitney U test. (C) As in (B) for cells transfected with mock miRNA, determined using RT-qPCR and calculated using the delta-delta Ct method. Significance of the difference between epithelial and mesenchymal marker gene expression was calculated using a one-sided Mann-Whitney U test. (D) Growth of parental (black) and resistant (red) cells under drug pressure at the corresponding IC50 of the parental cells after transfection with mock miRNA, relative to mock miRNA transfected cells unexposed to the drugs. Shown are data from individual experiments (points) and the means (bars). Significance of the difference between parental and resistant cell growth was calculated using a one-sided Mann-Whitney U test. For all cell line pairs, n = 6, except for PancVB, PancB1Q and PancB1V, n = 3, each replication consisting of 9 technical replicates. ns: non-significant, *: p-value< 0.05, **: p-value< 0.01, ***: p-value< 0.001, ****: p-value< 0.0001 [file 13148_2020_821_MOESM3_ESM.png]

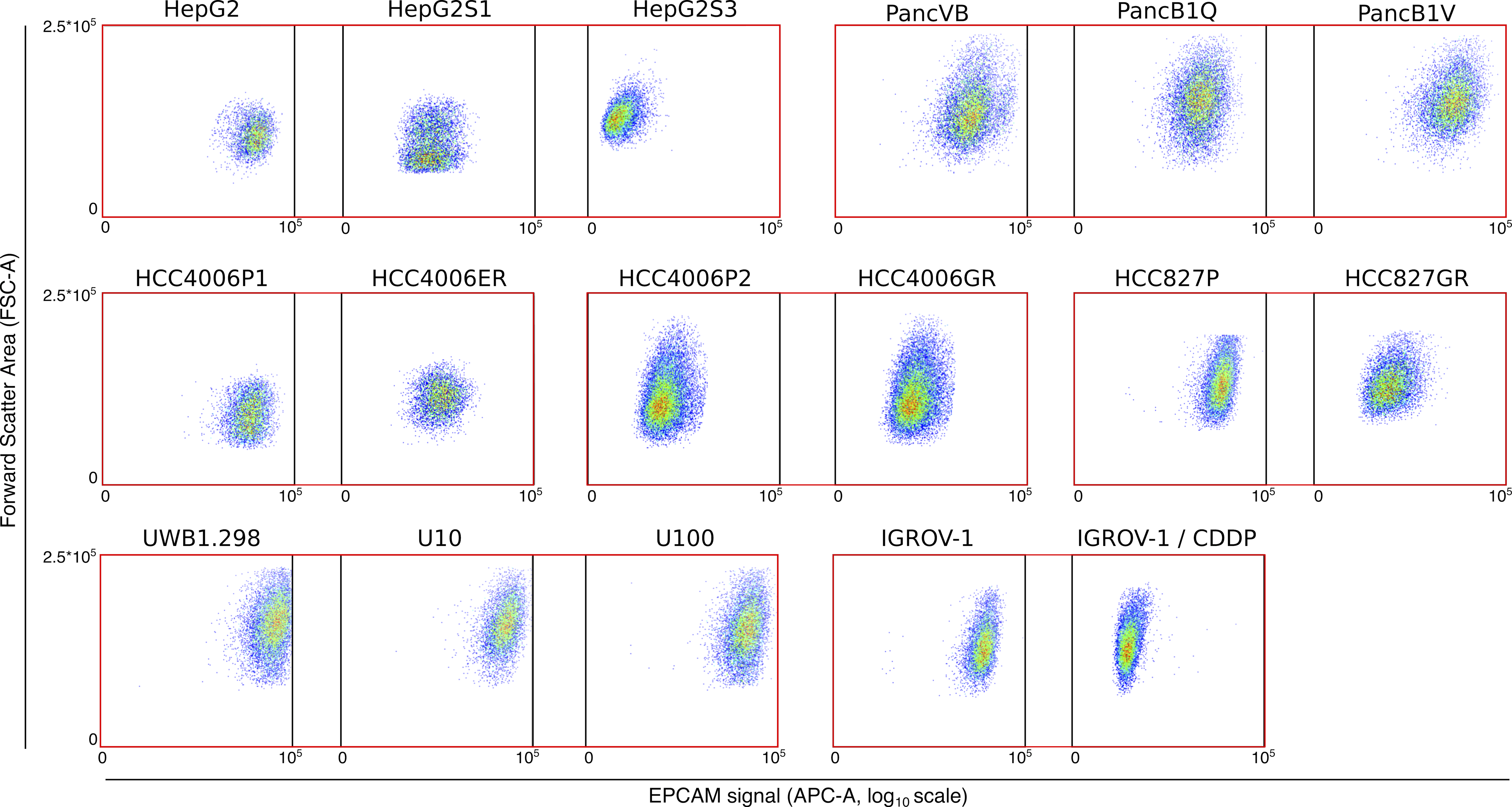

Supplement: Supplementary file 5 — Additional file 5:Figure S3. Cell surface expression of EPCAM protein in all cell lines. Shown are signal intensities of APC-conjugated anti-EPCAM antibody (x-axis, log10 scale) and Forward Scatter Area (FSC-A; y-axis, linear scale), assessed by flow cytometry. Red boxes contour data panels from cell lines originating from the same parental cell line. [file 13148_2020_821_MOESM5_ESM.png]

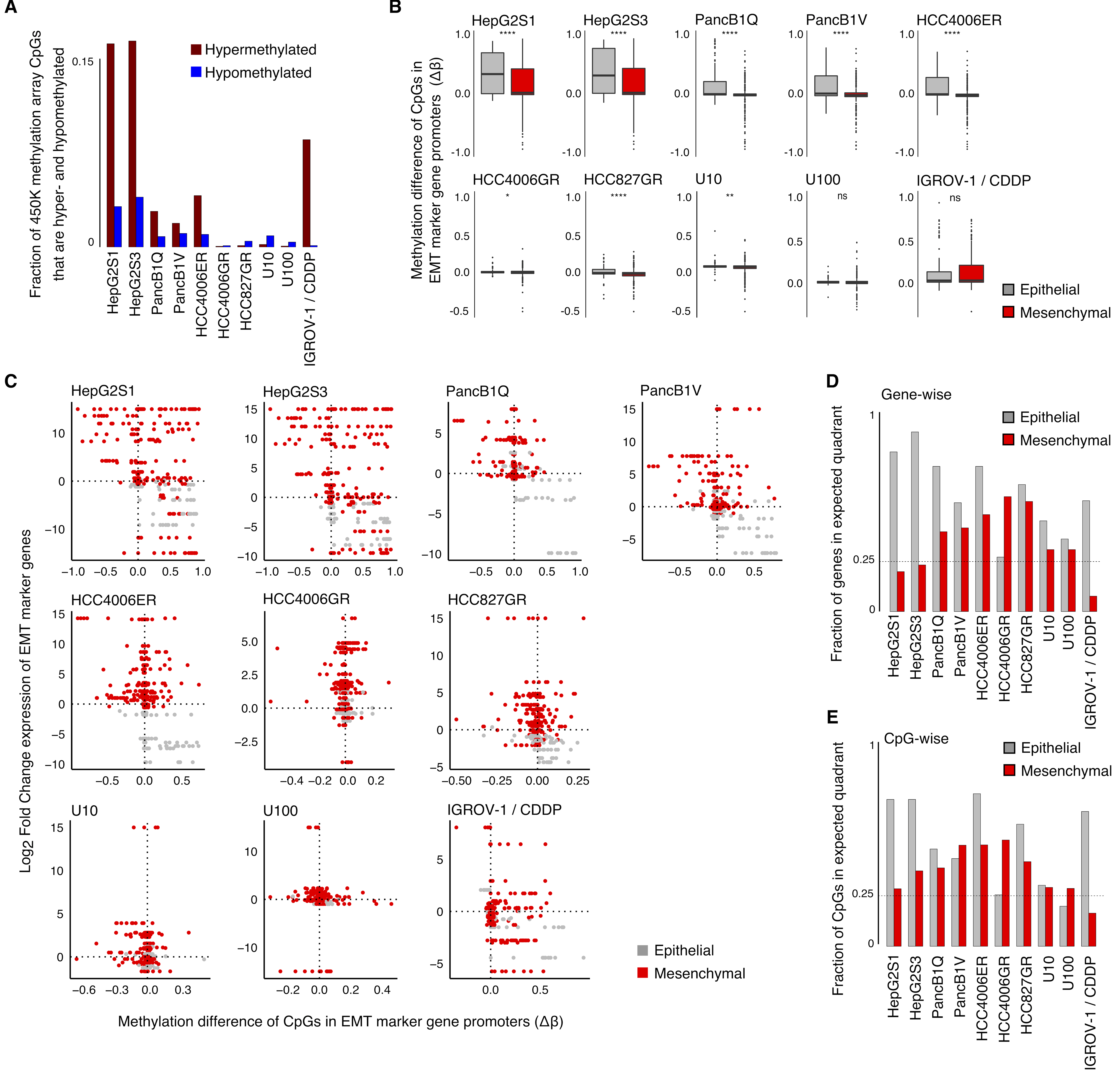

Supplement: Supplementary file 7 — Additional file 7: Figure S5. DNA methylation profiles correlate with EMT expression profiles (A) Fraction of hyper- and hypomethylated CpGs upon resistance acquirement. Red bar plots represent the fraction of interrogated CpGs (all CpGs included on the 450 K array) that are hypermethylated (with ßparental < 0.3 and ßresistant > 0.7) and blue bar plots the fraction of interrogated CpG that are hypomethylated (ßparental > 0.7 and ßresistant < 0.3) in resistant cells relative to parental cells. (B) Methylation changes upon resistance (Δß = ßresistant - ßparental) of CpGs in epithelial (117) and mesenchymal (275) marker gene promoter regions represented by gray and red boxplots respectively. Significance of the difference between epithelial and mesenchymal promoter methylation changes was calculated using a one-sided Mann-Whitney U test. (C) Correlation of EMT marker gene expression with promoter methylation. On the x-axis, the methylation difference of each CpG in EMT marker gene promoters between resistant and parental cells is depicted. On the y-axis, the log2 (fold-change) expression difference between resistant and parental cells of the corresponding EMT marker gene is depicted. In EMT models, mesenchymal gene promoter CpGs (red dots) are expected in the upper left quadrant and epithelial gene promoter CpGs (gray dots) in the lower right quadrant. (D) Fraction of epithelial (gray bar plots) and mesenchymal (red bar plots) genes that lie respectively in the lower right and upper left quadrant of Fig. 2b. The dotted line indicates the expected fraction (0.25). (E) Fraction of epithelial (gray bar plots) and mesenchymal (red bar plots) CpGs that lie respectively in the lower right and upper left quadrant of panel C. The dotted line indicates the expected fraction (0.25). ns: non-significant, *: p-value< 0.05, **: p-value< 0.01, ***: p-value< 0.001, ****: p-value< 0.0001. [file 13148_2020_821_MOESM7_ESM.png]

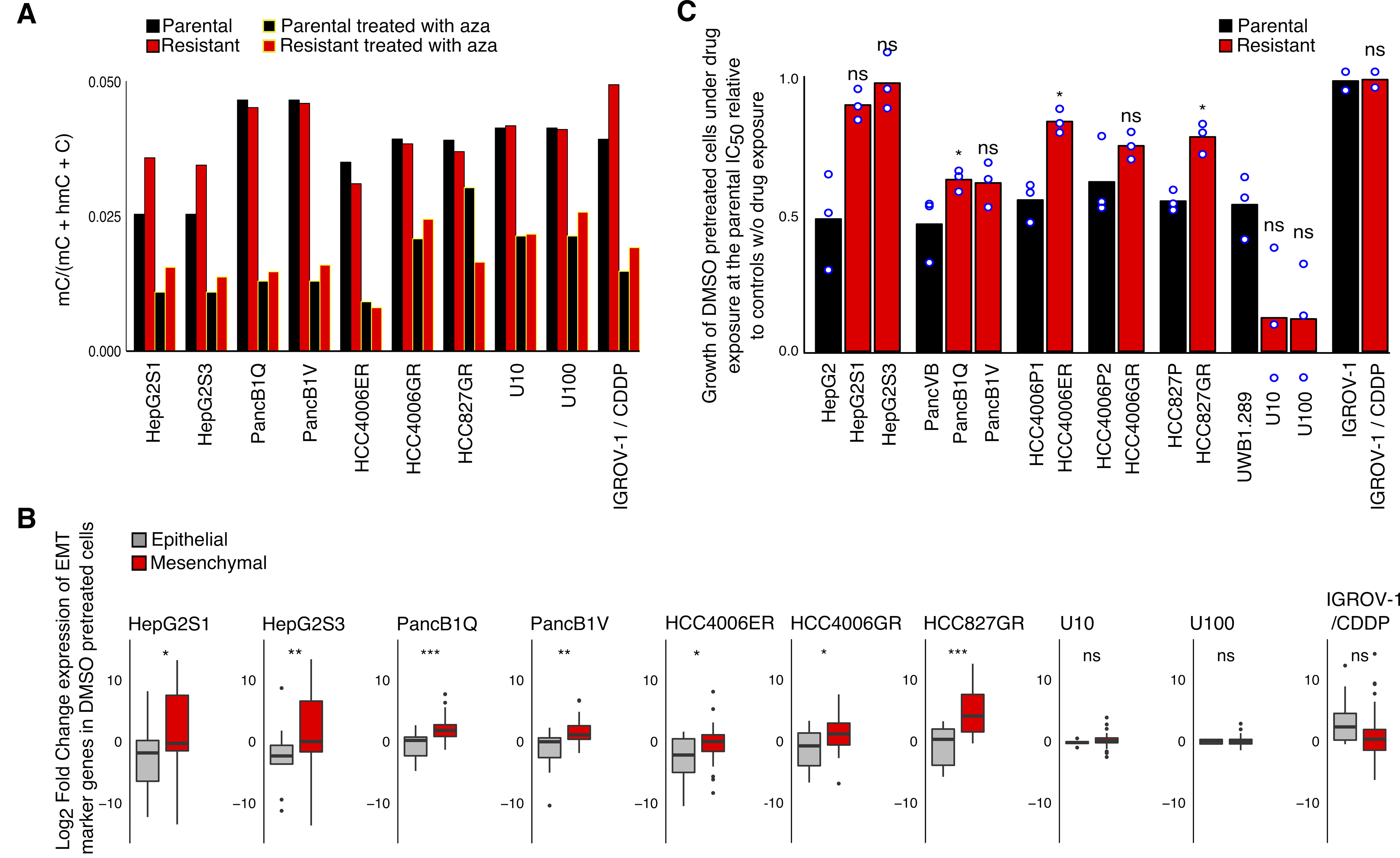

Supplement: Supplementary file 8 — Additional file 8: Figure S6. Control experiments concerning pharmacological demethylation of the cell line models. (A) Global cytosine methylation levels in parental (black) and resistant (red) cells determined by LC/MS before (no contour) and after (yellow contour) an 8-day exposure to a non-cytotoxic dose (0.5 μM) of the pharmacologically demethylating agent 5-aza-2′-deoxycytidine. Methylated cytosine levels are expressed relatively to all cytosines. Names of resistant lines are used to indicate the cell pairs. (B) EMT marker gene expression difference between resistant and parental cells after an 8-day vehicle (DMSO) treatment. Boxplots depict log2(fold-change) expression differences between resistant and parental cells of 16 epithelial (gray) and 34 mesenchymal (red) marker genes determined by RT-qPCR and calculated using the delta-delta Ct method. Significance of the difference between epithelial and mesenchymal gene expression changes was calculated using a one-sided Mann-Whitney U test. (C) Cell growth of parental (black) and resistant (red) cells after an 8-day vehicle (DMSO) treatment under drug pressure at the estimated IC50 of the parental cells (Primary data in Table 1) is depicted on the y-axis, expressed relative to growth of DMSO-pretreated cells not exposed to the drugs. Shown are data from individual experiments (points) and the means (bars). Experiments were performed in triplicate (except for IGROV-1/CDDP, n = 2), each replication consisting of 9 technical replicates. Significance of the difference between parental and resistant cell growth was calculated using a one-sided Mann-Whitney U test. ns: non-significant, *: p-value< 0.05, **: p-value< 0.01, ***: p-value< 0.001, ****: p-value< 0.0001. [file 13148_2020_821_MOESM8_ESM.png]

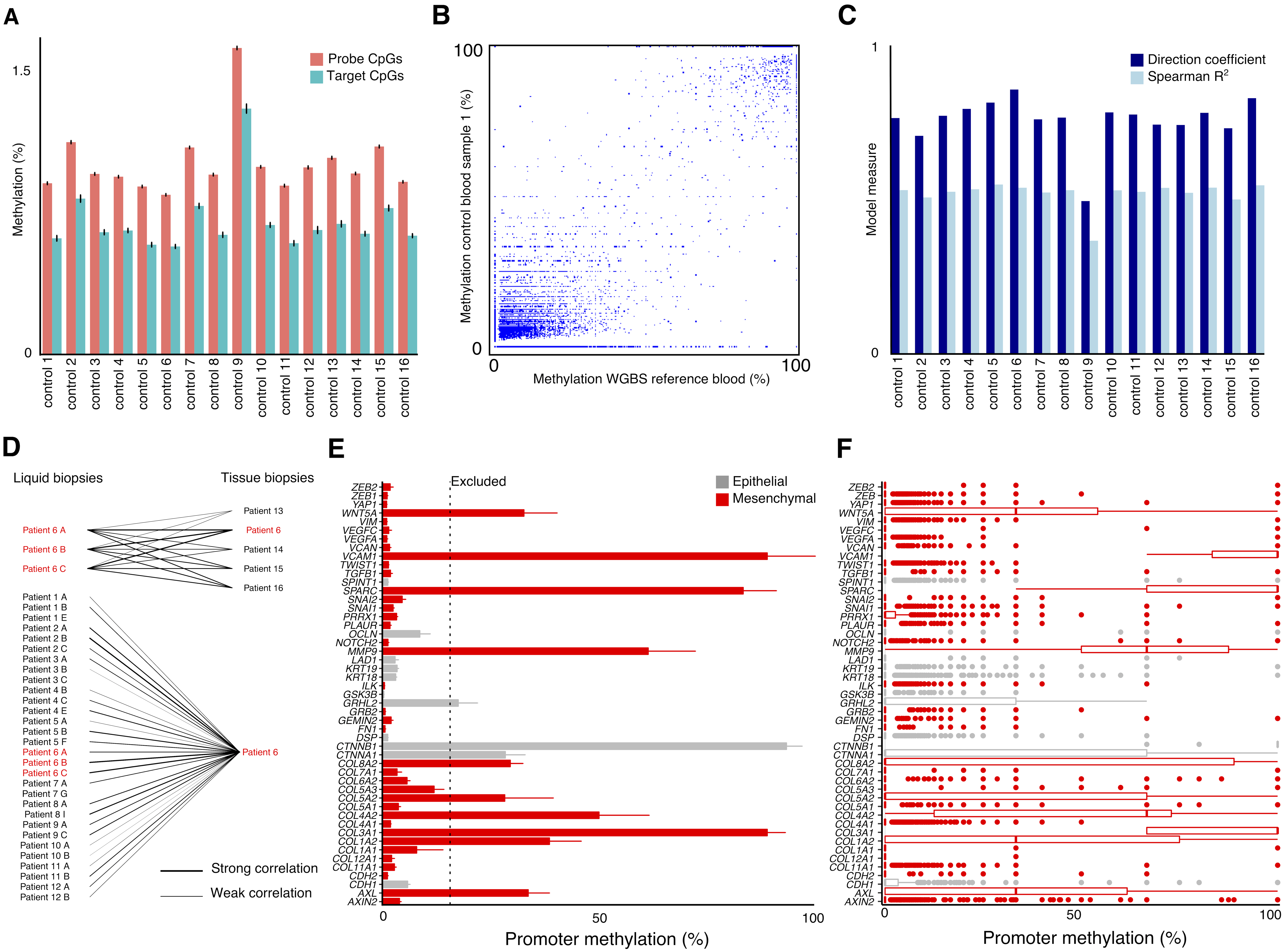

Supplement: Supplementary file 12 — Additional file 12:Figure S10. Methylation in control blood samples. (A) Bar plots depict methylation levels (%) of all CpGs included on the custom probes (in salmon) and of target CpGs (in green) for all 16 control samples. Shown are means ± SEM. (B) Probe CpG methylation levels (%) as measured by our novel technique in the first control blood sample (y-axis) plotted against published methylation levels of corresponding CpGs assessed in normal blood from a healthy male subject using WGBS (GEO accession number GSM1091963 [34], x-axis, %). (C) Linear model coefficients (direction coefficient and Spearman R2) of the correlation depicted in panel B for all control samples. (D) Pairwise correlation between tissue biopsies methylation results and the methylation results of the liquid biopsies. Thickness of the connection indicates the corresponding Spearman correlation R of the connected biopsies (Primary data available in (Additional file 1: Tables S7 and S8)). (E) For each EMT marker gene (y-axis), promoter methylation is calculated as the average methylation of all its CpGs (data pooled from all control samples) and depicted as bar plots (x-axis). Shown are means ± SEM. The dotted line indicates EMT marker genes that are considered too highly and/or variably methylated in normal blood and are therefore excluded for further analyses in this study. (F) Boxplots depict CpG methylation levels (%) of all EMT marker gene promoter regions, from all 16 control samples. [file 13148_2020_821_MOESM12_ESM.png]
